# Prematurity and genetic liability for autism spectrum disorder

**DOI:** 10.1186/s13073-025-01552-3

**Published:** 2025-10-02

**Authors:** Yali Zhang, Ashraf Yahia, Sven Sandin, Ulrika Åden, Kristiina Tammimies

**Affiliations:** 1 Center of Neurodevelopmental Disorders (KIND), Centre for Psychiatry Research, Department of Women’s and Children’s Health, Karolinska Institutet, Stockholm, Sweden; 2 Astrid Lindgren Children’s Hospital, Karolinska University Hospital, Region Stockholm, Stockholm, Sweden; 3 Department of Medical Epidemiology and Biostatistics, Karolinska Institutet, Stockholm, Sweden; 4 Department of Psychiatry, Icahn School of Medicine at Mount Sinai, New York, USA; 5 Seaver Center for Research and Treatment at Mount Sinai, New York, USA; 6 Department of Women’s and Children’s Health, Karolinska Institutet, Stockholm, Sweden; 7 Department of Neonatology, Division of Neonatal Medicine, Karolinska University Hospital, Stockholm, Sweden; 8 Department of Bioclinical Sciences, Linköping University, Linköping, Sweden

**Keywords:** Prematurity, Autism spectrum disorder, Genetics, Polygenic risk score, Machine learning, Generalized estimating equations model

## Abstract

**Background:**

Autism spectrum disorder (ASD) is a neurodevelopmental condition characterized by diverse presentations and a strong genetic component. Environmental factors, such as prematurity, have also been linked to increased liability for ASD, though the interaction between genetic predisposition and prematurity remains unclear. This study aims to investigate the impact of genetic liability and preterm birth on ASD conditions.

**Methods:**

We analyzed phenotype and genetic data from two large ASD cohorts, the Simons Foundation Powering Autism Research for Knowledge (SPARK) and Simons Simplex Collection (SSC), encompassing 78,559 individuals for phenotype analysis, 12,519 individuals with genome sequencing data, and 8104 individuals with exome sequencing data. Statistical significance of differences in clinical measures was evaluated between individuals with different ASD and preterm status. We assessed the rare variants burden using generalized estimating equations (GEE) models and polygenic load using the ASD-associated polygenic risk score (PRS). Furthermore, we developed a machine learning model to predict ASD in preterm children using phenotype and genetic features available at birth.

**Results:**

Individuals with both preterm birth and ASD exhibit more severe phenotypic outcomes despite similar levels of genetic liability for ASD across the term and preterm groups. Notably, preterm-ASD individuals showed an elevated rate of de novo variants identified in exome sequencing (GEE model, *p* = 0.005) in comparison to non-ASD-preterm group. Additionally, a GEE model showed that a higher ASD PRS, preterm birth, and male sex were positively associated with a higher predicted probability for ASD in SPARK, reaching a probability close to 90%. Lastly, we developed a machine learning model using phenotype and genetic features available at birth with limited predictive power (AUROC = 0.65).

**Conclusions:**

Preterm birth may exacerbate multimorbidity present in ASD, which was not due to ASD-associated genetic variants. However, increased ASD-associated rare variants may elevate the likelihood of a preterm child being diagnosed with ASD. Additionally, a polygenic load of ASD-associated variants had an additive role with preterm birth in the predicted probability for ASD, especially for boys. Future integration of genetic and phenotypic data in larger preterm or population-based cohorts will be crucial for advancing early ASD identification in preterm subgroup.

**Supplementary Information:**

The online version contains supplementary material available at 10.1186/s13073-025-01552-3.

## Background

Autism spectrum disorder (ASD) is an early-onset neurodevelopmental condition characterized by challenges in social interaction, communication, and restrictive and repetitive behaviors and interests [1]. In addition to these core symptoms, individuals with ASD have multiple co-occurring neurodevelopmental, psychiatric, and physical conditions, which contribute to clinical heterogeneity [2].

The etiology of ASD is multifaceted and not yet fully elucidated [3, 4]. However, genetic factors account for up to 80–90% of the liability for ASD [4–6]. Rare de novo variants (DNV), especially those affecting the gene function of constraint genes, are shown to be enriched in ASD [7, 8]. Rare inherited variants in ASD-related genes are also shown to be overtransmitted from parents to their children with ASD [5, 9]. In clinical genetic testing, the molecular diagnostic yield in ASD is ~ 7–40% depending on the phenotypic presentation [10]. In addition to rare variants, genome-wide association studies (GWAS) have identified a few common variants associated with ASD, and the polygenic load calculated using polygenic risk score (PRS) has demonstrated predictive ability for ASD and ASD traits [11, 12], explaining 2% of variance in ASD status [13]. Furthermore, ASD PRS can uniquely predict variability in cognitive performance [12].

In addition to genetic factors, there are several environmental factors associated with ASD [3]. The most robustly associated environmental stressor is prematurity, with ASD likelihood in preterm about two to four folds higher than in term, and ASD likelihood increasing as gestational age at birth decreases [14, 15]. Although preterm birth involves both genetic and environmental components [16], it is typically discussed as an environmental factor in ASD studies [3, 17]. Preterm birth, defined as delivery with gestational age before 37 weeks, can be further categorized into four preterm sub-categories: extremely preterm (< 28 weeks), very preterm (28–31 weeks), moderate preterm (32–33 weeks), and late preterm (34–36 weeks). Prematurity is not only associated with ASD but also with neurocognitive development and other health outcomes [18–20]. Earlier studies investigating phenotypes in children with ASD suggested that extremely or very preterm ASD children have more language deficits and developmental delays compared to term ASD children [21, 22]. However, others reported that no significant differences in development were found when studying the entire preterm group [23]. Moreover, preterm birth as an exposure is associated with various comorbidities in ASD, including attention and behavioral problems, neurological disorders, and growth deficiency. However, more investigations are needed to understand ASD phenotypic spectrum in preterm and term birth as well as how different sub-groups of prematurity contribute to specific medical outcomes and traits.

Interestingly, preterm infants have been found to have increased DNV rates compared to term [24], and various de novo copy number variations (CNVs) in preterm were found related to neurodevelopmental disorder-related genes (NDD genes) [25], but it remains uncertain whether DNV burden is further elevated when both ASD and preterm birth are present. Moreover, the relationship between ASD polygenic load and prematurity has only been evaluated by Cullen et al. based on cognition, but no interaction was found between ASD PRS and gestational age at birth [17]. Furthermore, there are indications that a small fraction of preterm individuals would have recognizable genetic disorders [26]. However, there have not been specific studies focusing on genetic factors within preterm individuals and ASD.

While genetic and phenotypic studies on the population level are informative, these analyses may miss interactions and non-linear relationships within or between factors on an individual level. To address this complexity, machine learning (ML) has the potential to identify patterns in high-dimensional data that traditional statistical methods may overlook, aiding in prediction. To date, ML prediction models have emerged to predict ASD using different data sources, such as routine medical assessments and electronic records [27, 28], genetic data [29], and integrative models [30]. However, none of the published ML models currently predict ASD in preterm children. In the existing ML models for ASD prediction, included features are typically collected when the child is at least 1–2 years of age or older [27, 31]. It remains unclear whether integrating phenotype and genetic information available at birth could enable earlier ASD identification in preterm infants.

In this study, we aimed to enhance our understanding of ASD in preterm children by analyzing both clinical and genetic data in two large ASD cohorts, the Simons Foundation Powering Autism Research for Knowledge (SPARK) [8, 32], and the Simons Simplex Collection (SSC) [33]. Across individuals with different ASD and prematurity sub-groups, we first examined their phenotype severity through the prevalence and multimorbidity of other medical diagnoses. Thereafter, we assessed the burden of rare and common sequence-level variants. Finally, we built an ML model using both phenotype and genetic features that could be obtained at birth to predict ASD in preterm individuals.

## Methods

### Study cohorts

This study utilized the Simons Foundation Powering Autism Research for Knowledge (SPARK) cohort [32] for the main analyses and the Simons Simplex Collection (SSC) cohort [33] for validation. Both cohorts are initiated by the Simons Foundation Autism Research Initiative (SFARI), with recruitment of individuals with autism spectrum disorder (ASD) and families living in the USA. Phenotypic information (medical and psychiatric diagnosis, quantitative measures) and genotype data (genome and exome sequencing) were obtained through SFARI Base (https://sfari.org/sfari-base) [34] and previous published studies [8, 35, 36]. The exact sample source and sample size for each analysis part are described below in the corresponding sections and in Table 1. All procedures adhered to the ethical standards of the Helsinki Declaration, with informed consent obtained from all participants and legal guardians or parents for participants unable to provide consent themselves.

**Table 1:** Characteristics of the analyzed participants

		Cohort		SPARK (version 9)		SSC
				**ASD**	**Non-ASD**	**ASD**
**Phenotype analysis**		Number of samples		74,217	/	1636
		Preterm in ASD (%)		12.4%	/	9.6%
		Preterm		9196	2706	157
			Extremely preterm (GA < 28 weeks)	699 (8%)	109 (4%)	/
			Very preterm (GA 28–31 weeks)	987 (11%)	274 (10%)	2 (1%)
			Moderate preterm (GA 32–33 weeks)	1274 (14%)	372 (14%)	11 (7%)
			Late preterm (GA 34–36 weeks)	5659 (62%)	1754 (65%)	144 (92%)
			Unknown GA	577 (6%)	197 (7%)	/
			Male:Female	6860:2336	1358:1348	139:18
		Term		65,021	/	1479
			Male:Female	48,069:16,952	/	1270:209
**Genetic analysis**	De novo variant (WGS)	Preterm		309 (10.2%)	164	137 (9.4%)
			Male:Female	257:52	90:74	121:16
		Term		2728	/	1313
			Male:Female	2172:556	/	1129:184
	De novo variant (WES)	Preterm		697 (10.8%)	210	/
			Male:Female	563:134	119:91	/
		Term		5747	/	/
			Male:Female	4557:1190	/	/
	Inherited variant	Preterm		310 (10.2%)	165	/
			Male:Female	258:52	90:75	/
		Term		2742	/	/
			Male:Female	2182:560	/	/
	PRS	Preterm		305 (10.1%)	161	155 (9.7%)
			Male:Female	252:53	86:75	137:18
		Term		2702	6472	1435
			Male:Female	2134:568	2817:3655	1236:199
**Machine learning**		Preterm		279	150	/
			Male:Female	230:49	81:69	/
		Term		2601	1,664	/
			Male:Female	2064:537	799:865	/

/ Mark as data were not analyzed. *GA* Gestational age at birth. The % number labeled after sample size of preterm in each analysis represents the frequency of preterm birth among ASD individuals in that analysis part

### Phenotypic data processing

We utilized demographic and phenotype data from the SPARK collection version 9 with a release date 2022–12-12. We considered medical and psychiatric diagnosis history from the basic medical screen dataset, grouping specific diagnoses into nine diagnostic categories: behavior, development, mood, growth, birth, eating habits (Eat), neurological conditions (Neuro), visual and auditory impairments (Visaud), and sleep. This dataset includes 9196 individuals with ASD and born preterm with gestational age less than 36 weeks (ASD-preterm), 65,021 individuals with ASD and born term (ASD-term), and 2706 individuals without ASD and born preterm (non-ASD-preterm). We also stratified preterm individuals into four sub-groups based on gestational age: extremely preterm (< 28 weeks), very preterm (28–31 weeks), moderate preterm (32–33 weeks), and late preterm (34–36 weeks). Additionally, we compared quantitative measures using the Child Behavior Checklist (CBCL) [37] *t*-score for 1 to 5 and 6 to 18 years of age, Developmental Coordination Disorder Questionnaire (DCDQ) [38] final score, Repetitive Behavior Scale-Revised (RBS-R) [39] total final score, Social Communication Questionnaire (SCQ) [40] final score, and Full-Scale Intelligence quotient (FSIQ) [41] score. Additional file 1: Tables S1 and S2 provide detailed description of the specific diagnoses and quantitative measures.

For the SSC cohort, due to the absence of preterm information for non-ASD individuals (siblings of ASD probands), we only conducted studies for ASD-preterm and ASD-term groups. After excluding individuals with unreliable gestational age, unknown ASD diagnosis, births occurring post-term (gestational age > 40 weeks), and missing outcome information, we retained 1637 probands diagnosed with ASD (157 preterm and 1479 term) for the diagnostic category analysis. Additionally, we analyzed available quantitative measures, including CBCL score [37], DCDQ score [38], SCQ score [40], and IQ score [41]. Detailed descriptions of specific diagnoses and qualitative measures are provided in Additional file 1: Tables S3 and S4.

### Genotype data source

For the genetic part, dataset SPARK genome sequencing (GS) version 1.1 variant calling was used, including 12,519 individuals from 3394 families, with 315 ASD-preterm, 2788 ASD-term, and 155 non-ASD-preterm individuals. In SPARK, DNA was extracted from saliva samples, and sequencing was conducted on the Illumina NovaSeq 6000 system. Variant calling was performed using GATK (v3.5) with HaplotypeCaller, and all samples were jointly called by GLnexus (v1.4.1). We also utilized earlier published DNV data from exome sequencing (ES) to calculate the event rate [8]. These DNVs were called by DeepVariant (v.0.8.0). Among the 6444 ASD individuals with DNV information, 5747 were born full-term, and 697 were born preterm. Furthermore, DNV information was accessible for 210 preterm children without ASD.

For the SSC cohort, we incorporated the de novo variants (DNV) dataset from Ng et al. in our analysis, encompassing 1450 ASD individuals after excluding post-term births [35]. DNA extracted from blood, GS was conducted on the Illumina HiSeq X Ten platform, and DNVs were called by GATK (v4.1.0) and DeepVariant (v0.10). Additionally, we obtained the Polygenic Risk Score (PRS) dataset from Weiner et al. [36], with scores generated using the Ricopili pipeline. This dataset comprised 1590 ASD individuals, excluding post-term births.

### De novo variant calling and analysis

To find de novo variants (DNV) of children, we included all trios in the SPARK GS version 1.1 dataset. For families with more than one child, each child forms a trio with their parents, resulting in multiple trios within the same family. There were 5712 trios from 3364 families. We used two tools, Slivar (v0.2.8) and GATK (v4.1.4.1), to call the DNV from SPARK trios, and true DNV was selected when it was found in both tools [42, 43]. The DNV was called if it was labeled as “denovo” with allele balance (AB) in children higher than 0.25 in Slivar and identified as high-confidence DNV in GATK. For pseudo-autosomal regions on the sex chromosome, we separately considered the variant genotype as 1/0 in children. We did not find DNVs on chrY in pseudo-autosomal regions. Then, we did quality control to further filter the rare DNV by removing variants with GQ < 20, DP < 10, gnomAD population allele frequencies > 0.001, and variants of either 10 A’s or T’s in a row. We filtered out DNVs on genomic centromeres and low complex regions. Then we removed DNV if (1) it can be found in other family’s parents, (2) it can be found in only children but more than three families, and (3) it on positions having more than 3 multi-alleles. We identified 432,903 DNVs, including 16,155 exonic DNVs and 986 loss-of-function (LOF) variants. We filtered out 29 children with DNV counts beyond three times the standard deviation from the mean DNV count. Based on these criteria, the average number of rare DNVs per child was 75.9.

We annotated DNVs by ANNOVAR (v2020.06.08) with buildver as hg38 and protocol as ljb26_all, SnpEff (v5.2) with GRCh38.105 databases, and VEP (v110) with assembly as GRCh38 and 154 dbSNP [44–46]. DNVs with Sequence Ontology (SO) terms as “frameshift”, “splice_acceptor”, “splice_donor”, “start_lost”, “stop_gained”, and “stop_lost” in gene effect were considered as LOF DNV. Additionally, we identified variants on the neurodevelopmental disorder-related genes (NDD gene) using high-confidence ASD genes collected by the Simons Foundation Autism Research Initiative (SFARI) (2024–01-16 release) with gene scores of 1 or 2 and labeled as syndromic and green gene list of Intellectual disability—microarray and sequencing (Version 5.497) on Genomics England PanelApp (2024–03-14 accessed) (Additional file 2) [47, 48].

### Inherited variants calling

Using the same SPARK GS data as for DNV calling, including 5712 trios from 3364 families, we extracted variants on the genomic protein-coding NDD genes (Additional file 2) using VCFtools (v0.1.16) [49]. Then we annotated the inheritance mode of NDD genes using the ID gene panel app, SysNDD database (v0.1.0) and DDgenes [48, 50, 51]. There were 80% (1625 genes) of NDD genes annotated, including 738 dominant genes coded as monoallelic or dominant, and 944 recessive genes coded as biallelic or recessive in databases (Additional file 2). To restrict the analysis to rare inherited variants, we used the allele frequency filter threshold of 0.001 and 0.01 for dominant and recessive genes, respectively. Variants with GQ < 20, DP < 10, and genotypes conflicting with the inheritance mode of located genes were filtered out. We did not find compound variants (more than 1 heterozygous variant on the same recessive gene for one child). For variants on dominant genes, we identified LOF following the same process in the DNV part and found damaging missense variants met at least one of the following conditions: CADD > = 20, SIFT labeled as D, POLYPHEN labeled as P and D, PHYLOP > = 2.0 or REVEL > = 0.5. From 5712 trios in the SPARK GS database, we identified 245,671 and 4346 inherited variants in dominant and recessive NDD genes, respectively. We identified 2717 LOF and 39,136 damaging missense variants in genes with a dominant inheritance mode.

### Polygenic risk score

Based on GS jointly called variants from 12,519 SPARK participants, we performed quality control for individuals and variants using PLINK1.9 with parameters listed in Additional file 1: Table S5, retaining 11,933 individuals and 9,558,997 variants with a total genotyping rate of 99.9% [52]. Then, genome coordinates of variants were converted from hg38 to hg19 using liftOver (v2017-03–14) [53], and 9,428,216 were mapped after removing duplicated variants. We calculated the posterior SNP effect size estimates using PRS-CS with ASD GWAS summary statistics from the Psychiatric Genomics Consortium (November 2017 release) (46,351 individuals) and European LD reference data from 1000 Genome phase III [11, 54]. The default parameters used in PRS-CS also be listed in Additional file 1: Table S5. The final PRS was calculated using the score function in PLINK1.9 with the estimated posterior SNP effect size. To minimize the effects on different populations, we analyzed the ancestry of individuals using principal component analysis (PCA) by pca command in PLINK1.9. The ten principal components (PC1-10) were included as covariates when we calculated the association between phenotype and PRS.

### Statistical analysis

All analyses were performed using R programming language (v4.2.2). We compared two pairs of groups: preterm and term birth within individuals diagnosed with ASD (ASD-preterm versus ASD-term), and individuals diagnosed with ASD to those without ASD within preterm birth (ASD-preterm versus non-ASD-preterm).

In phenotypic analysis, an individual was considered to possess the diagnosis feature if any specific diagnosis within that diagnostic category (Additional file 1: Table S1) was present, regardless of the number of specific diagnoses present simultaneously. The prevalence is reported by the frequency of individuals with the diagnostic categories. We examined the differences in prevalence between ASD-preterm and ASD-term, ASD-preterm and non-ASD-preterm using odds ratios (ORs) with 95% confidence interval (CI) and statistical significance reported by FDR-adjusted *p*-values. After stratifying preterm stages, we used *χ*2 test to evaluate differences across preterm stages, post hoc comparisons for each pair of preterm stages, and Kendall’s tau test to examine rank correlation of preterm stages with prevalences. Then, prevalences and ORs of multimorbidity (one, two, three, four, or not less than five diagnostic categories; Additional file 1: Tables S1) were estimated by ASD and preterm status, and prevalence differences in multigroup were examined by *χ*2 test. We also examined prevalence and multimorbidity ORs adjusted for sex and age using generalized estimating equation (GEE) models, with family ID as a clustering variable. GEE model is more robust to assumptions of data following a particular data distribution and adjusts for correlations between individuals, e.g., siblings and families [55]. Additionally, we examined differences in quantitative measures using the 2-sided Wilcoxon rank sum test for pairwise comparisons and in multiple comparisons using the Kruskal–Wallis rank sum test. To account for multiple testing, we applied false discovery rate correction to *p*-values.

The burden of DNV and inherited variants was evaluated by comparing rates of such variants in each subgroup categorized by ASD and preterm status. We assessed the statistical differences between groups through GEE models with Poisson family, sex as a covariate, and family ID as a clustering variable. PRSs were *z*-standardized and statistical significance for PRS distribution was reported by the 2-sided Wilcoxon rank sum test. The association between targeted phenotype (y/n) and PRS in each subgroup was also evaluated in GEE logistic model with sex (m/f) and PC1-10 from ancestry checking as covariates.

The variance of ASD status explained by PRS in the SPARK cohort was quantified using the McFadden Pseudo-*r*^2^ value based on the logistic regression model adjusted for sex and ancestry PC1-10. To examine the associations between ASD diagnosis and possible variables, we modeled the probability of ASD (y/n) by fitting GEE logistic model(s) with the equation as [ASD (y/n) ~ sex (m/f) + preterm (y/n) + standardized PRS] and family ID as a clustering variable in European population to find the correlation between ASD diagnosis and possible variables. To visualize the predicted probabilities of ASD from the GEE logistic model, we utilized ggemmeans function in ggeffects R package (v1.5.1) [56], showing the average predicted probabilities of ASD for specific levels of variables adjusted for other covariates in the model. After that, the variable (preterm (y/n) * standardized PRS) was added to the GEE logistic model to check the correlation between ASD (y/n) and the interaction of preterm status and PRS. To detect the association between multimorbidity and DNV burden, GEE models and linear regression were used.

### Machine learning model

This part of the analysis was performed in R (v4.2.2). We utilized non-ASD and ASD diagnoses as two classification flags, incorporating features obtained from data that can be collected at birth. For phenotypical variables, we included birth complications, sex, and birth-related conditions. For term individuals without a recorded gestational age, a value of 40 weeks was imputed. Genetic variables encompassed the count of several types of genetic variants, the CADD score of de novo variants (DNV), and the PRS of ASD. To remove redundant variables and identify informative features, we employed the Recursive Feature Elimination (RFE) algorithm [57]. RFE is a feature selection technique that iteratively removes the least important features based on model performance, refitting the model with the remaining features until the optimal subset of features is identified. In RFE, we utilized random forest function and tenfold cross-validation in the underlying model to assess feature importance throughout the process. For the features selected after RFE, we only retained the more general feature (e.g., retaining LOF over LOF on NDD genes) in any pair of features with a correlation coefficient above 0.7 to reduce multicollinearity. The details of selected features are listed in Additional file 1: Table S6.

We applied R package caret (v6.0–94) [58] to train the ML models. Given the limited sample size and the higher proportion of ASD samples than non-ASD, we conducted nested cross-validation (NCV) and hyperparameter tuning with grid search to enhance model performance. In NCV, we partitioned the data into 10 folds in the outer loop, with nine folds used for training and the remaining fold for testing. Within the inner loop, we performed repeated 10*tenfold cross-validation, and the model with the best performance was applied to the outer loop. We employed three algorithms—Extreme Gradient Boosting (XGBoost), Random Forest (RF), and Linear Support Vector Machine (SVM)—to construct the models. The values of hyperparameter tuning for each model are detailed in Additional file 1: Table S7. We reported evaluation metrics, including accuracy with a 95% CI, area under the receiver operating characteristic curve (AUROC), specificity, sensitivity, and F1-score. Moreover, the SHapley Additive exPlanations (SHAP) values for features were computed and visualized using the R package SHAPforxgboost (v0.1.3) [59], quantifying the contribution of each feature to individual model predictions in terms of direction and magnitude.

## Result

### Phenotype comparison across ASD and prematurity

**Fig. 1:**
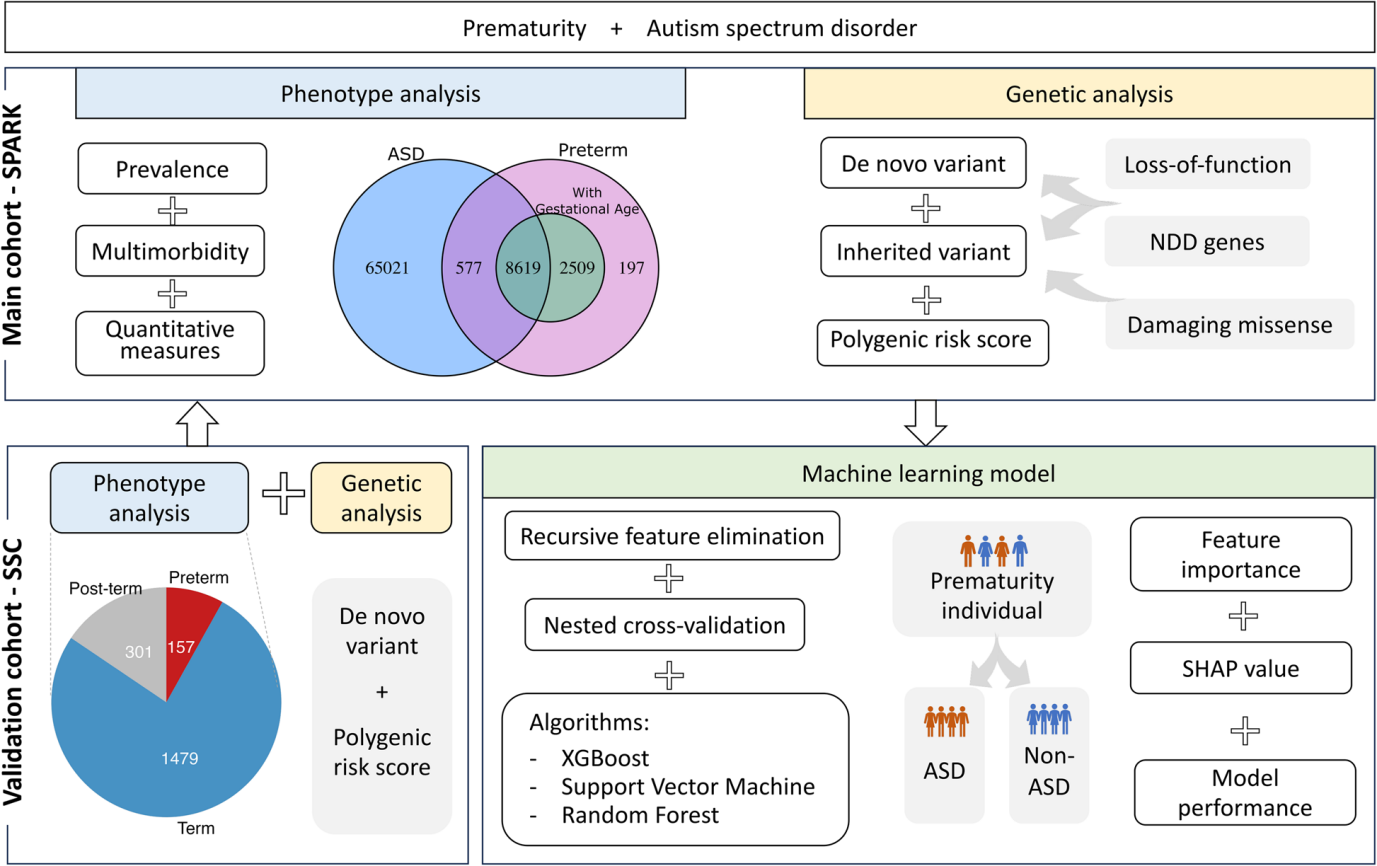
Overview design of the study. Firstly, we performed phenotype analysis on diagnosis prevalence, burden of multimorbidity and quantitative measures in the SPARK cohort. The sample size of SPARK is shown in the Venn diagram, with blue indicating ASD, pink indicating preterm with unknown gestational age, and green indicating preterm with known gestational age. Secondly, we analyzed the de novo variant and inherited variant burden, separately, focusing on loss-of-function variants and damaging missense and if these affected neurodevelopmental disorder (NDD) genes. Additionally, we utilized polygenic risk scores for common variants associated with ASD. For validation, we applied similar analyses in the SSC cohort. Thirdly, we integrated phenotype and genomic data to train the machine learning models with different algorithms to predict ASD diagnosis in the preterm group. Shapley additive explanations (SHAP) values assess the effect of each feature on the model performance

We utilized basic medical screening data from 181,248 individuals in the SPARK version 9 cohort (release date 2022–12-12). Among them, 74,217 (41%) were diagnosed with ASD, and 11,902 (7%) were born preterm. When performing phenotype comparison, we grouped individuals with 9196 individuals with ASD and being preterm (ASD-preterm), 65,021 individuals with ASD but being term (ASD-term), and 2706 preterm individuals without ASD diagnosis (non-ASD-preterm) (Fig. 1, Table 1). In the SSC cohort, gestational age records were available only for probands with ASD, of which 157 were preterm and 1479 were term. We stratified the preterm stage based on gestational age at birth (Table 1).

**Fig. 2:**
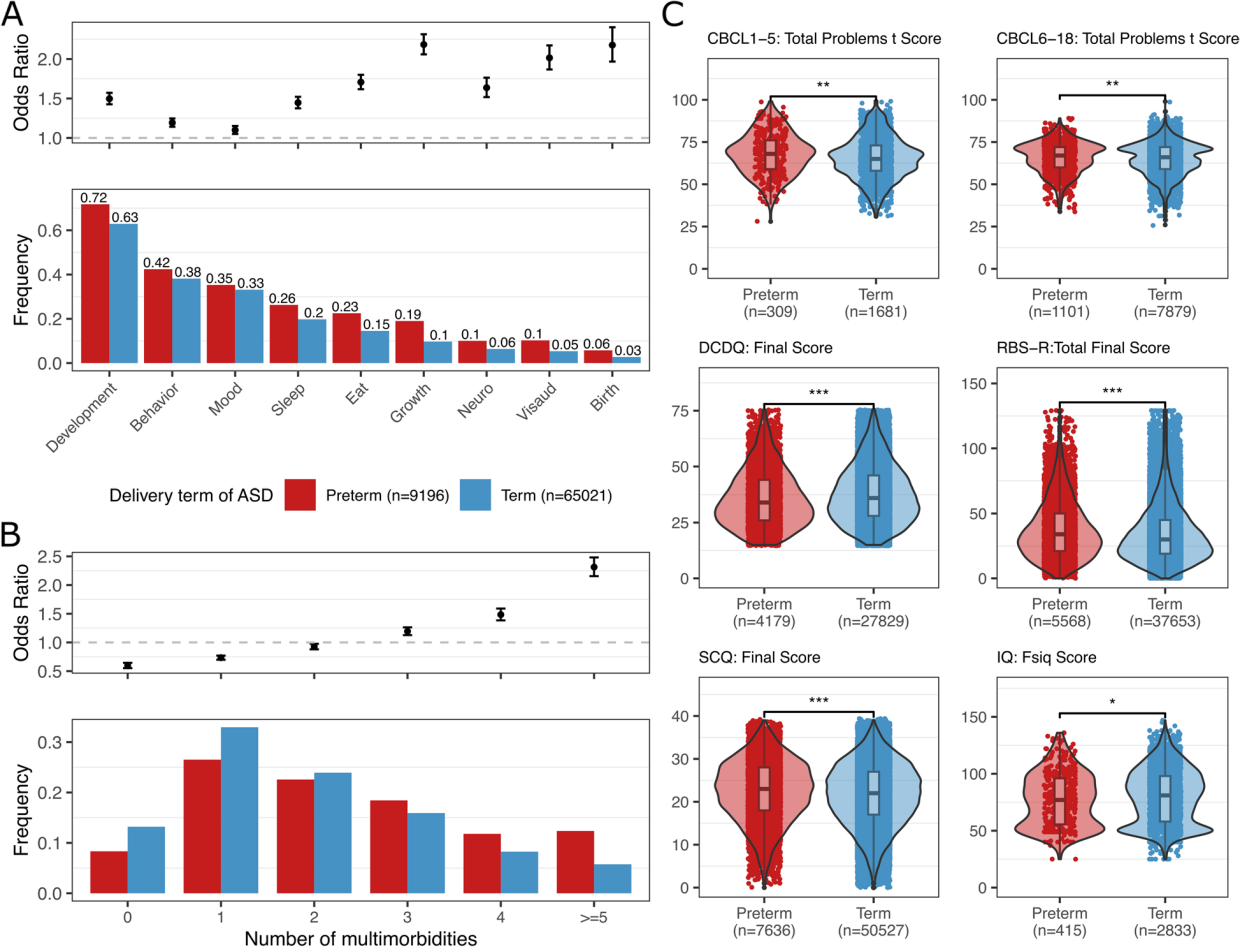
The phenotype comparison between preterm and term with ASD in the SPARK version 9 cohort. Color bars are the same across three panels and shown at the bottom of panel **A**. **A** Prevalence and odds ratio with 95% confidence interval (CI) of diagnostic categories. The detailed outcomes in each diagnostic category are described in Additional file 1: Table S1. The exact prevalence values are labeled on the top of the bars. ORs are given among ASD individuals born preterm vs term and recorded in Additional file 1: Table S8. **B** Distribution of the number of multimorbidities, defined as the total number of diagnostic categories (development, behavior, mood, sleep, eat, growth, neuro, visaud, and birth; same as in **A** and described in Additional file 1: Table S1) that appear in individuals. ORs with 95% CI are given among ASD individuals born preterm vs term when focusing on each multimorbidity number and recorded in Additional file 1: Table S10. **C** Differences in Child Behavior Checklist (CBCL) *t*-score for 1 to 5 and 6 to 18 years of age, Developmental Coordination Disorder Questionnaire (DCDQ), Repetitive Behavior Scale-Revised (RBS-R) score, Social Communication Questionnaire (SCQ), and Full-Scale IQ (Fsiq) among ASD individuals born preterm and term. Significance was assessed using the 2-sided Wilcoxon rank sum test with the FDR-adjusted *p*-values marked in the plots as 0–0.001***, 0.001–0.01**, 0.01–0.05*, or NS (non-significant difference)

We performed analysis on nine available diagnostic categories recorded in the basic medical screening dataset in SPARK phenotype database version 9, involving behavior, development, mood, growth, birth, eating habits (eat), neurological conditions (neuro), visual and auditory impairments (visual), and sleep (details of diagnostic categories are described in Additional file 1: Table S1). For each category, we assigned a binary variable indicating the presence or absence of conditions within that category, rather than counting the number of specific diagnoses. The prevalence of all diagnostic categories analyzed was higher in ASD-preterm compared to ASD-term (Fig. 2A). Specifically, ASD-preterm had higher odds ratio (OR) for all diagnostic categories, with the highest being for birth and growth diagnoses (ORs = 2.18 [95% CI 1.97–2.40] and 2.18 [95% CI 2.06–2.31], respectively). Additionally, preterm birth was associated with a modestly increased likelihood of other behavioral diagnoses compared to term in the ASD group (OR = 1.19 [95% CI 1.14–1.25]). The results remained consistent after adjusting for sex, age, and family linkage using GEE models (Additional file 1: Table S8). We also observed significantly different prevalences of diagnostic categories (*χ*^2^ test with FDR-adjusted *p* < 0.001 for all categories) when we considered different sub-groups of preterm birth (Additional file 1: Fig. S1A). Furthermore, we identified linear trends across different preterm stages, with groups of lower gestational age showing a higher prevalence in growth, eating, neuro, and visual diagnostic categories (Kendall’s tau test, FDR-adjusted *p* = 0.04). Almost all the preterm sub-groups had a higher prevalence of diagnostic categories compared to the term stage (FDR-adjusted *p* of the post hoc comparisons of *χ*^2^ test are in Additional file 1: Table S9).

Then, we analyzed multimorbidity, indicated as the number of concurrent diagnostic categories (Additional file 1: Table S1) among the ASD individuals, revealing that ASD-preterm exhibited a higher likelihood of the higher number of concurrent morbidities compared to ASD-term (Fig. 2B, *χ*^2^ test with *p* < 2.2 × 10^−16^; ORs are recorded in Additional file 1: Table S10). In ASD, preterm sub-groups showed differences in the burden of multimorbidity (Additional file 1: Fig. S1B, *χ*^2^ test with *p* < 2.2 × 10^−16^), in which extremely and moderate preterm subgroups exhibited a significantly higher burden of multimorbidity with ≥ 5 diagnoses compared to late preterm (Additional file 1: Table S11, post hoc test of *χ*^2^ test with FDR-adjusted *p* = 1.6 × 10^−5^, 0.02 respectively). For diagnostic categories with more than two specific diagnoses, we found a positive linear correlation between the number of specific diagnoses, i.e., multimorbidity, and the odds ratio of preterm versus term, except for birth-related issues (Additional file 1: Fig. S1C). This indicated that preterm ASD individuals tend to have more specific diagnoses within categories as well as across categories than term ASD individuals.

Next, we analyzed quantitative measures for overall behavioral challenges and specific symptom domains. We observed significant differences between ASD-preterm and ASD-term (2-sided Wilcoxon rank sum test with FDR adjustment), although the large sample size may amplify the differences (Fig. 2C). ASD-preterm had increased severity of behavioral challenges (CBCL score for 1–5 years, *p* = 1.4 × 10^−3^; for 6–18 years, *p* = 0.0043), developmental coordination disorder (DCDQ final score, *p* = 2.5 × 10^−21^), repetitive behaviors (RBS-R score, *p* = 6.7 × 10^−34^), and social communication skills (SCQ score, *p* = 1.4 × 10^−20^), as well as lower IQ scores (*p* = 0.02). Comparing different sub-groups of preterm birth, we found that extremely preterm has the lowest DCDQ final score compared to other stages (2-sided Wilcoxon rank sum test, FDR-adjusted *p*-values are 0.003, 0.002, and 2.7 × 10^−5^ when compared to very preterm, moderate preterm, and late preterm, respectively) (Additional file 1: Fig. S2).

To complement our analyses within the ASD individuals, we analyzed if there were any differences within preterm birth for the same phenotype measures. The ASD-preterm had more severe outcomes in comparison to non-ASD-preterm with increased severity with lower gestational age (Additional file 1: Fig. S3A-B, S4). The developmental diagnostic category had the highest prevalence (72%) in the ASD-preterm group, resulting in 8.8 OR (95% CI 7.9–9.7) when compared to non-ASD-preterm (Additional file 1: Table S12). Also, ASD-preterm group was seven times as likely (OR = 7.0, 95% CI 5.4–9.4) to have five or more multimorbidities than non-ASD-preterm group (Additional file 1: Table S13). For quantitative measures, the ASD-preterm group had statistically significantly higher SCQ final scores compared to the non-ASD-preterm group (2-sided Wilcoxon rank sum test, *p* < 2.2 × 10^−16^) (Additional file 1: Fig. S3C).

ASD-preterm and term comparisons within the SSC cohort also showed a higher prevalence of eating and growth problems (OR = 2.58 [95% CI 1.79–3.68], and 2.02 [95% CI 1.06–3.58] respectively; Additional file 1: Table S14), and a similar trend towards having more multimorbidity compared to ASD-term (Additional file 1: Fig. S5A-B, *χ*^2^ test *p* = 0.045; Additional file 1: Table S15). No statistically significant differences were observed in the quantitative measures (Additional file 1: Fig. S5C).

### Genetic variants comparison across ASD and prematurity

**Fig. 3:**
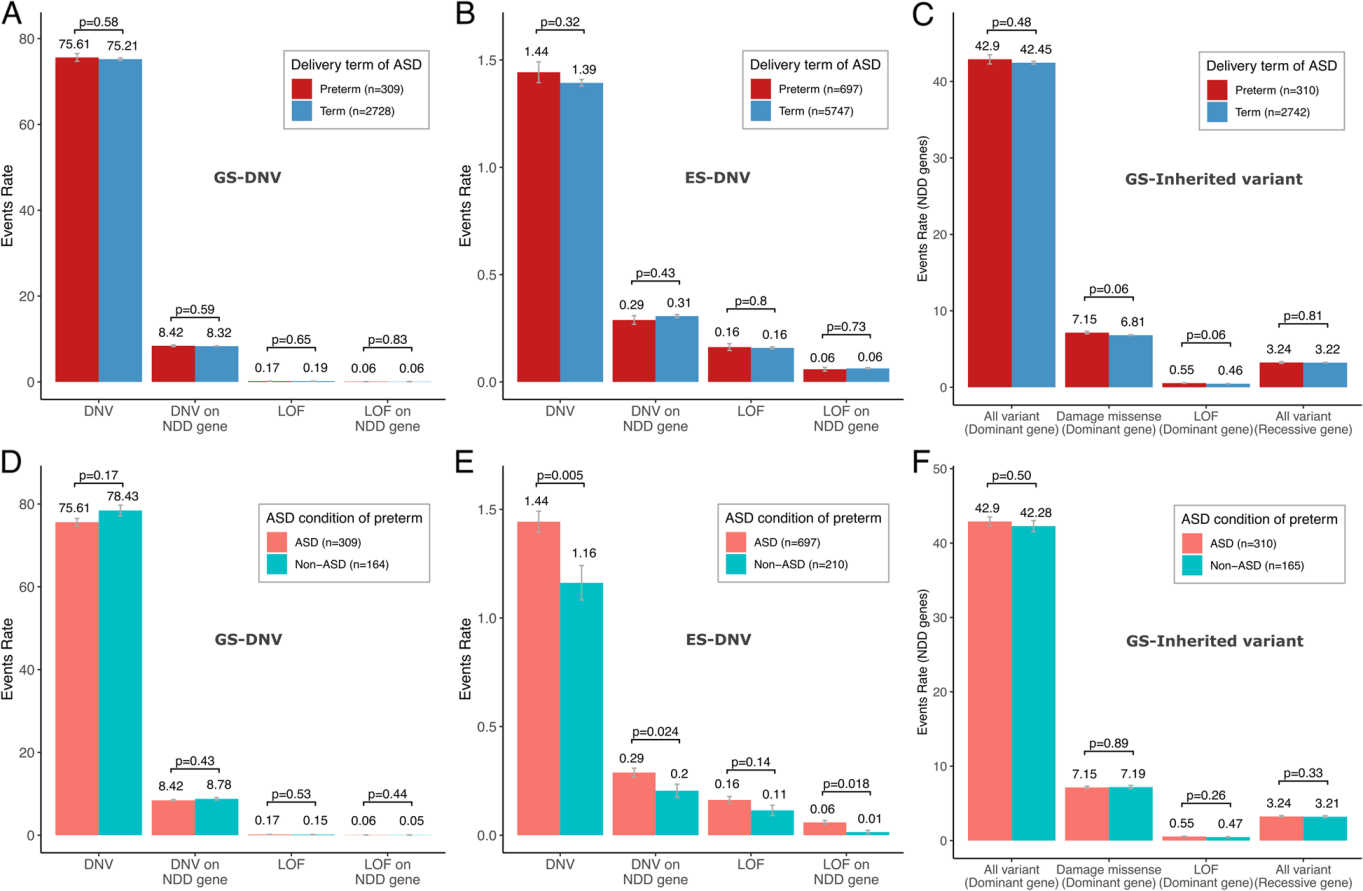
Association between genetic variant burden and subgroups with varying preterm and ASD status in SPARK. In SPARK cohort, for ASD individuals, event rates of rare de novo variants (DNV) identified through genome sequencing (**A**) and exome sequencing (**B**), and inherited variants on dominant and recessive NDD genes identified through genome sequencing (**C**) were calculated. In preterm individuals, event rates of de novo variants identified through genome sequencing (**D**) and exome sequencing (**E**), and inherited variants in dominant and recessive NDD genes identified through genome sequencing (**F**) were calculated. Rare DNVs were filtered with AF < 0.001, and rare inherited variants were filtered with AF < 0.001 on dominant genes and AF < 0.01 on recessive genes. Data are presented as mean values ± standard errors as error bars. The GEE model with Poisson family and sex covariate was used to compute the *p*-values to assess the differences in variant count between groups

To investigate the burden of de novo variants, we analyzed available genome sequencing (GS) and exome sequencing (ES) data from SPARK and SSC. The population analyzed for GS included 310 ASD-preterm, 2742 ASD-term, and 165 non-ASD-preterm individuals. The ES dataset contained 697 ASD-preterm, 5747 ASD-term, and 210 non-ASD-preterm individuals. We did not observe any significant difference in DNV event rate or distribution of DNV numbers between ASD-preterm and ASD-term (Fig. 3A, Additional file 1: Fig. S6A), or between ASD-preterm and non-ASD-preterm derived from GS (Fig. 3D, Additional file 1: Fig. S6B), even when analyzing only the exonic region (Additional file 1: Fig. S7A-B). For the simplex family subset, no differences were observed between preterm and term ASD probands (Additional file 1: Fig. S8A). Similarly, no statistically significant differences were found comparing de novo burden from GS in 137 ASD-preterm and 1313 ASD-term in the SSC (Additional file 1: Fig. S7C).

When analyzing DNV event rates obtained from ES data [8] from SPARK, no statistically significant differences were found between ASD-preterm and ASD-term (Fig. 3B, Additional file 1: Fig. S6C), even when restricted to probands in simplex families (Additional file 1: Fig. S8B). However, ASD-preterm individuals had more exonic DNVs (*p* = 0.005), exonic DNVs on NDD genes (*p* = 0.024), and LOF affecting NDD genes (*p* = 0.018) than non-ASD-preterm (Fig. 3E, Additional file 1: Fig. S6D). We stratified the ASD-preterm and non-ASD-preterm by gestational age preterm subgroups and observed a similar trend in both the moderate and late preterm groups. However, due to the limited sample size, a statistically significant difference between ASD-preterm and non-ASD-preterm was only detected in the event rate of DNVs in NDD genes in the moderate preterm group (*p* = 0.02) and in both overall DNV and DNV in NDD genes in the late preterm group (*p* = 0.002 and 0.04, respectively). Interestingly, for extremely to very preterm stages, the event rates of DNV were numerically lower in ASD compared to non-ASD individuals, even though this difference was not statistically significant (Additional file 1: Fig. S9).

We also investigated the rates of inherited variants, focusing on those affecting NDD genes and protein-coding regions. From 4974 individuals with phenotype information, we did not observe statistically significant differences between ASD-preterm and ASD-term nor between ASD-preterm and non-ASD-preterm, although ASD-preterm tends to have a numerically higher rate of rare inherited variants (Fig. 3C, F, Additional file 1: Fig. S6E, S6F). No differences were observed between preterm and term ASD probands in simplex families as well (Additional file 1: Fig. S8C).

To further evaluate whether the greater multimorbidity is associated with increased DNV burden, and whether multimorbidity and DNV burden are jointly associated with the increased likelihood of ASD and preterm, we computed GEE models (Additional file 1: Table S16) and found that multimorbidity is positively correlated with GS LOF (*p* = 0.037), GS LOF on NDD genes (*p* = 5.3 × 10^−06^) and all types of ES DNV burden (*p* = 1.1 × 10^−^^05^, 1.9 × 10^−09^, 6.6 × 10^−14^ and < 2.2 × 10^−6^ for DNV, LOF, DNV on NDD genes and LOF on NDD genes respectively) across all individuals. Stratified by preterm and ASD status, we observed this positive correlation pattern in ASD-term group for ES DNV (*p* = 0.009), ES DNV on NDD genes (*p* = 0.013), and LOF on NDD genes (*p* = 0.004), as well as in ASD-preterm group for ES LOF on NDD genes (*p* = 0.013) (Additional file 1: Fig. S10). However, except for GS DNV on NDD genes, there is no interaction between DNV burden and multimorbidity performing on ASD or preterm outcomes.

### ASD polygenic risk score and association with preterm status

**Fig. 4:**
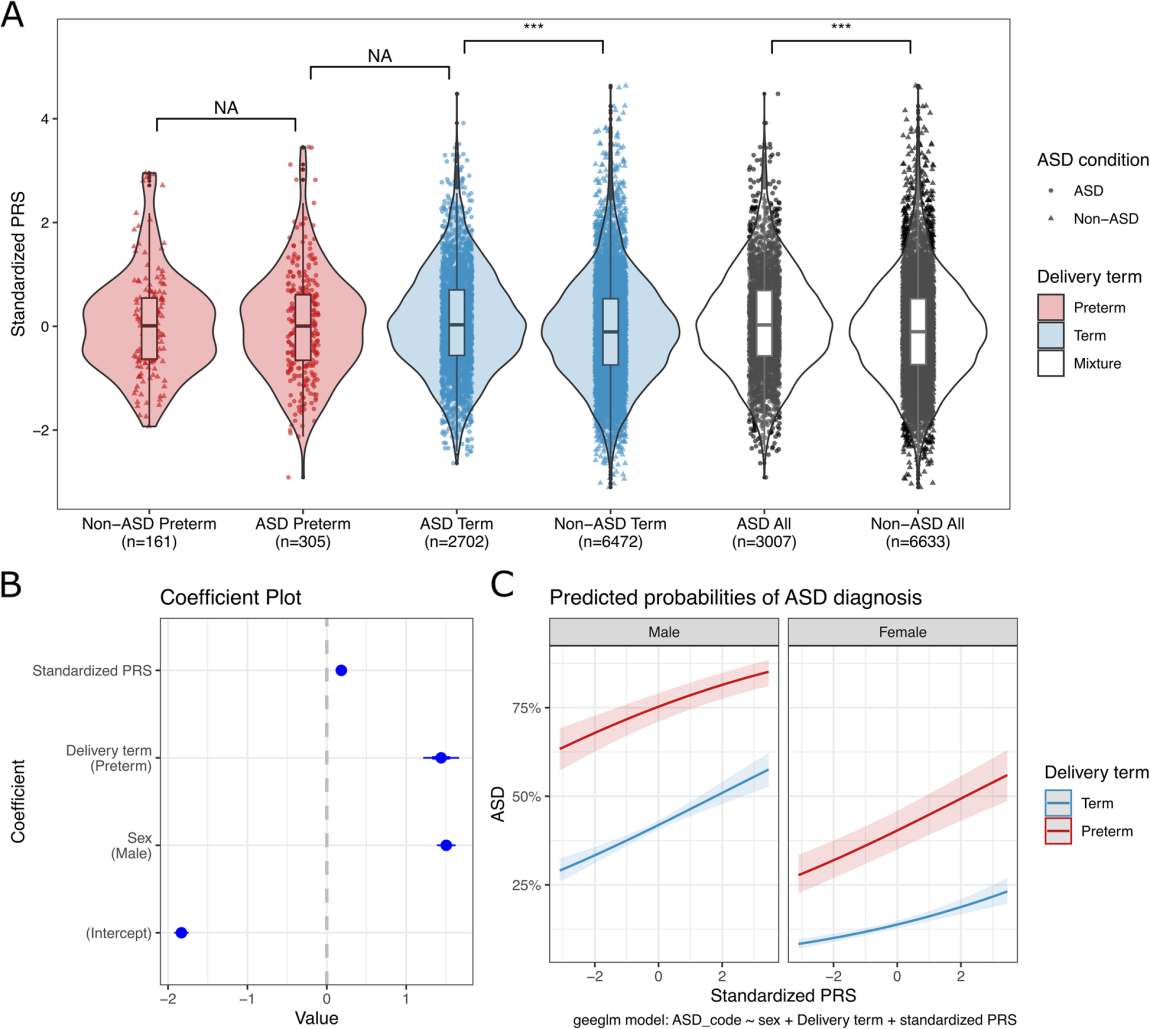
The association of polygenic risk score (PRS) with delivery term and ASD condition in SPARK.** A** The distribution of standardized PRS in groups with different delivery terms and ASD diagnosis. Statistical significance was assessed using the 2-sided Wilcoxon rank sum test with the *p*-value marked in the plots as 0–0.001*** or NS (statistically non-significant difference). **B** Coefficient plot for the GEE logistic model [ASD(y/n) ~ sex(m/f) + Preterm status(y/n) + Standardized PRS] with family ID as a clustering variable, displaying the estimated coefficients for each variable. Positive coefficients suggest an increase in the likelihood of ASD associated with the variable, while negative coefficients indicate a decrease. Error bars represent 95% CI. **C** Visualized effect plot of GEE model, which shows average predicted probabilities of ASD diagnosis in SPARK for specific levels of variables, with color region around the line showing 95% CI

We calculated ASD PRS for individuals in the SPARK cohort using the most comprehensive GWAS on ASD as source data [11]. There was no significant difference in the distribution of PRS between the ASD-preterm and ASD-term groups (even restricted to simplex family probands) nor between ASD-preterm and non-ASD-preterm (Fig. 4A). As expected, ASD individuals had higher PRS compared to non-ASD individuals in the whole cohort displaying the usability of the PRS (2-sided Wilcoxon rank sum test, *p* = 6.7 × 10^−13^) (Fig. 4A). The PRS explained 9.8% (McFadden Pseudo-*r*^2^) variance of ASD diagnosis in the SPARK cohort. Additionally, after adjusting for sex and population ancestry (as indicated by principal components [PC]) in a GEE logistic model, we confirmed that there was no independent association between preterm birth and PRS (in ASD population), or between ASD diagnosis and PRS (in preterm population). The statistically non-significant association between preterm birth and PRS (in ASD population) was replicated in the SSC cohort, and same for simplex family probands in SPARK (Additional file 1: Fig. S11). Furthermore, we computed a full GEE logistic model for ASD diagnosis within European populations, showing that male sex, preterm birth, and higher PRS were all positively associated with ASD diagnosis (*p* < 2 × 10^−16^, < 2 × 10^−16^, and 2.2 × 10^−12^, respectively) (Fig. 4B). In this model for the SPARK cohort, the predicted probability of an ASD diagnosis was almost 90% for preterm-born males, with the highest PRS (Fig. 4C). Then, we included the interaction between preterm status and PRS in the full GEE logistic model, observing a significant association between this interaction and ASD diagnosis (*p* = 0.017).

### Predictive model for ASD within preterm births

Lastly, we investigated the potential of ML models to identify those preterm infants with a high likelihood of ASD from information present at birth by combining clinical and available genetic data. The model was developed and tested using a study population with preterm individuals classified into ASD (*n* = 279) and non-ASD (*n* = 150). For features used in the prediction model, we also considered Combined Annotation Dependent Depletion (CADD) scores, which assess the potential impact (i.e., deleterious or benign) of genetic variants on the function of genes and were available for most of the DNVs. We applied Recursive Feature Elimination (RFE) and a correlation threshold of 0.7 to select 13 features, including clinical features (sex, condition of birth complications, gestational age, insufficient oxygen at birth) and genomic features (number of several types of variants, CADD scores and standardized ASD-PRS) (Additional file 1: Fig. S12A, Table S6). We used three algorithms to train the models (Table 2), of which the XGBoost model exhibited the highest area under the receiver operating characteristic curve (AUROC), at 0.65. The model accurately identified 69% (95% CI 0.644–0.733) ASD diagnoses in the preterm, with a sensitivity of 0.81, specificity of 0.47, and F1-score of 0.77. We also trained a model using the same set of features on all individuals, including both preterm and term (assuming a gestational age of 40 weeks for terms), and despite the sample size (1814 non-ASD and 2880 ASD) being approximately 10 times larger than that of the preterm model, the model performance was comparable (XGBoost with accuracy = 0.69 [95% CI 0.672–0.699]; AUC = 0.68; F1-score = 0.76; Table 2).

**Table 2:** Performance metrics of machine learning models for ASD prediction in SPARK and preterm individuals

Population	Algorithm	Accuracy	95% CI	AUC	Sensitivity	Specificity	F1-score
**Preterm individuals**	XGBoost	0.69	(0.64, 0.73)	0.65	0.81	0.47	0.77
	Random forest	0.67	(0.63, 0.72)	0.63	0.86	0.33	0.77
	SVM	0.70	(0.66, 0.75)	0.62	0.84	0.46	0.79
**All SPARK individuals**	XGBoost	0.69	(0.67, 0.70)	0.68	0.80	0.52	0.76
	Random forest	0.68	(0.67, 0.69)	0.66	0.82	0.44	0.76
	SVM	0.70	(0.68, 0.71)	0.65	0.80	0.52	0.76

The first three XGBoost models within the 10-fold training (Additional file 1: Fig. S12B) were selected to visualize feature effects for the best-performing model for ASD within preterm births. The feature importance varied slightly across the training XGBoost models with sex, PRS, and CADD score being the most important features (Additional file 1: Fig. S12C, S12E, S12G). Using SHAP values to characterize the impact of each feature on the model’s output for specific individuals, we found that sex had the highest significant impact on the model’s predictions, whereas being male had a positive impact on the model’s prediction. Furthermore, we demonstrate that lower gestational age, more autosomal exonic DNVs, more dominant inherited variants, more LOF variants, the presence of birth complications, and insufficient oxygen at birth drove the model towards ASD prediction result (Additional file 1: Fig. S12D, S12F, S12H).

## Discussion

Here, we conducted a comprehensive analysis of phenotypic differences using larger cohorts, as well as genotypic differences, which have been explored in only a few studies among preterm and term-birth ASD individuals. We conclude that preterm-born ASD individuals have more diagnoses across different categories and a number of co-occurring diagnoses but similar genetic landscapes when investigating sequence-level rare DNVs and inherited variants as well as a polygenic load for ASD compared with ASD-term. Our analysis of preterm individuals with and without ASD showed similar results for the phenotype comparisons but inconsistent findings for the genetic burden. The largest de novo dataset derived from ES showed that the ASD-preterm had a higher exonic DNV event rate than the non-ASD-preterm; however, we did not validate this finding in the de novo dataset from GS. Additionally, the male with preterm status and higher polygenic load faces a higher likelihood of ASD when considering these features together. Furthermore, our ML model demonstrated potential for predicting ASD diagnosis in preterm children by integrating phenotype and genetic information. Our results provide evidence that genetic factors play a role in emerging ASD in preterm birth, but the environmental stressor of being preterm potentially contributes to the severity and multimorbidity.

Previous research has reported numerous but inconsistent findings regarding phenotypic disparities between ASD preterm and term individuals [21, 23], while limited research has focused on investigating the genetic link of ASD in prematurity. Unlike most phenotypic comparisons that concentrate on specific diagnostic outcomes [22, 23], we first grouped the various conditions into nine broader diagnostic categories. Our results indicate that children with both preterm birth and ASD exhibit a higher prevalence of diagnoses within these categories and a higher rate of multimorbidity across different diagnostic categories. To note, we did not replicate all the observed associations in SSC cohort, which had mostly late preterm category for the GA and more restrictive recruitment criteria, which could explain the lack of replication. Previous studies have found that both preterm birth and ASD are associated with adverse symptoms. For instance, preterm infants are independently inherently prone to multimorbidity and severe health complications affecting multiple organs and systems [18, 60, 61], such as visual and auditory impairments [62], epilepsy [63], ADHD [19], and other psychiatric disorders [20]. Additionally, individuals with ASD experience a higher burden of co-occurring medical conditions [64]. Our study further supports the hypothesis that preterm acts as an environmental liability factor, potentially influencing some of the heterogeneity and higher comorbidity rates observed in ASD.

After stratifying preterm based on gestational age, we observed that those born with lower gestational age tend to have more severe outcomes, which is in line with the dose–effect reported in prematurity, where the likelihood of developmental issues increases with decreasing gestational age [61]. This effect is also reflected in the potentially increasing complexity of multimorbidity among groups with lower gestational age [18]. Additionally, we showed significantly more severe symptom levels, as measured by different standardized questionnaires and cognitive tests, in ASD-preterm, consistent with previous studies as well as general research comparing preterm and term birth [19, 65–67]. It is important to note that with large sample sizes, even very small differences can become statistically significant. In SSC cohort, with over 90% of preterm individuals being late preterm, thus replication of results was limited. Therefore, the results should be interpreted with caution especially for clinical utility.

In idiopathic ASD, heritability is estimated to be approximately 80% [6], but in preterm born, environmental factors account for 60% of the variation in gestational age [68]. Our findings suggest that genetic factors underlie, at least partly, the ASD diagnosis even in preterm, but that the complex phenotypic presentation, including multimorbidity, could be due to the environmental stressor of being preterm. Specifically, we did not observe significant differences in DNV numbers between preterm and term ASD individuals. We did observe suggestive evidence that DNV burden could be higher in ASD-preterm compared with non-ASD-preterm; however, the finding was inconsistent in the validation cohort with a smaller sample size and after stratifying by preterm stages. We speculate that this may be indicative of distinct underlying genetic mechanisms for ASD across different preterm sub-groups. Across preterm, in light of previous reports showing that ASD has a higher DNV burden than non-ASD [7], we suspect that some DNVs specifically affect ASD liability but not prematurity, or some DNVs could be liability factors for both ASD and prematurity. Limited research indicated a higher DNV burden in overall preterm newborn genomes and primarily in genes related to embryonic brain development; however, the study did not consider ASD or another behavioral diagnosis in preterm infants [24], especially ASD also susceptibility to brain development-related genes [69]. For the lack of differences in DNV burden observed between preterm and term in ASD, one hypothesis is that some DNVs contributing to ASD have global effects on overall physiology and also contribute to prematurity. In this situation, those DNVs could possibly increase the likelihood of both preterm birth and ASD with multimorbidity. Furthermore, it remains to be clarified to what extent prematurity acts as an environmental liability factor to ASD when counting in genetic background.

Although GWAS studies of prematurity have identified variations in maternal and fetal genes separately [70, 71], few have examined the impact of rare inherited variants. Our study did not find a difference in the burden of rare inherited variants between ASD-preterm and ASD-term individuals. This can be partially explained by the fact that the maternal genome influences prematurity more than the fetal genome [16]. Additionally, we have perhaps overlooked shared genetic factors that potentially contribute to both preterm birth and neurodevelopment when we analyze individuals with either ASD or prematurity. For example, variants at *AGTR2* and *ADCY5* genes in mothers have been associated with gestational duration and preterm birth, and infants show around half of effect size by sharing one of the maternal allele by descent [72]. Notably, these two genes are also known ASD-associated genes [47].

Although we did not find an overall association between ASD PRS and prematurity, we show intriguing findings that in the SPARK cohort, those with the highest PRS could have a higher likelihood of ASD, especially in preterm infants and boys. Even after including the interaction between preterm status and PRS, these features maintained a significant association with ASD likelihood, and the interaction itself was significantly associated with ASD diagnosis. The lowest PRS in preterm males still reached a 75% predicted probability of ASD, emphasizing the prematurity effect on ASD liability and the greater susceptibility in males, while also indicating a potential sample bias towards ASD in our analysis. Again, these findings need validation in the general population, especially as a prior study by Cullen et al. found no evidence of an interaction effect between ASD polygenic score and gestational age at birth on cognition [17]. However, it is important to note that the cognitive difficulty they measured is only one of the outcomes that do not imply an ASD diagnosis, and the model they used also included socio-economic status as a covariate.

In addition to genetic factors, widespread alterations in brain development associated with preterm infants may contribute to the increase in ASD likelihood. Previous studies have indicated that reduced structural brain asymmetry and poor brain development during neonatal life may increase the liability of ASD in preterm infants [73, 74]. Even in preterm children exhibiting similar ASD traits during childhood, distinct etiological trajectories have been observed involving variations in neonatal cerebellar volume and developmental delay [75].

Not all preterm infants develop ASD [76], still we demonstrate that when genetic factors are combined with the environmental risk of preterm birth, preterm children face a highly elevated likelihood of ASD diagnosis. Recognizing the limitations of traditional statistical models in capturing nonlinear interactions between features, we developed an ML model to predict ASD diagnosis in preterm children at birth. Unlike previous ASD prediction models that rely on developmental trajectories or typical characteristics collected as children grow [27, 31, 77], our ML model utilized only information available at birth, integrating phenotype and genetic information. Moreover, most previous models are based on the general population [27, 77], limiting their applicability to preterm infants. However, it is necessary to build prediction models tailored specifically for preterm infants due to the heterogeneity of ASD phenotypes [1], and preterm ASD children may exhibit specific phenotypes compared to term ASD children [20]. Although our ML model did not achieve significantly higher performance, achieving 69% accuracy with a small sample size and few features demonstrates the feasibility and efficacy of integrating phenotype and genetic information for ASD prediction. There is still substantial room for improvement in the model performance. Increasing the sample size and adding more features associated with preterm birth and ASD (e.g., maternal age, prenatal exposure, and fetal birth weight) would benefit the prediction model construction. Other features like intubation in the delivery room, family language, parental education, infection, and ventilation have also been found to have predictive ability for cognitive outcomes in very preterm infants [31]. It is important to note that we cannot identify the causal relationships between features selected by the model and ASD diagnosis. Still, we suggest that these features could potentially enhance prediction models in the future. As clinical genetic testing is recommended for all individuals with ASD [78], the recommendation could be expanded to preterm infants even prior to an ASD diagnosis.

Our study has several limitations. Firstly, two cohorts are ASD-focused, and the non-ASD groups are mainly relatives of ASD probands, thus introducing sample bias to ASD and potentially underestimating genetic differences between the groups. Given the high heritability estimation in ASD, siblings with a closer relationship with ASD have a higher relative risk ratio for ASD [4]. Secondly, we did not stratify the analyses by sex due to the limited sample size, potentially overlooking sex-specific differences. Thirdly, we focused here only on the sequence level variation, incorporating more types of genetic variations will be the next step. Furthermore, our exploration of genetic factors primarily focused on average population-level associations and NDD genes, potentially overlooking genetic effects beyond the currently known ASD-associated genes and variants that may contribute to the elevated likelihood of ASD in preterm children. Previous studies have pointed out the genetic association between preterm and ASD, such as common genetic variants linking abnormalities in the gut-brain axis with both conditions [79]. Additionally, the lack of comparability of SSC with the SPARK cohort may reflect both reduced statistical power and cohort differences. The SPARK cohort collected participants with different family structures and highly heterogeneous clinical severity. While the SSC cohort with a smaller sample size excluded probands with conditions might reduce the validity of diagnoses (e.g., known genetic syndromes, significant perinatal complications, and low mental age). In particular, the distribution of gestational age differs in two cohorts, and more than 90% of preterm in SSC are late preterm. Also, the sample size is low for some of the analyzed groups despite similar percentages of preterm in ASD in the two cohorts. Finally, our prediction model illustrates that integrating genetic and clinical information could aid in ASD liability assessment for preterm children. However, due to the lack of independent preterm cohorts with sufficient ASD individuals with genetic and clinical data, validation is limited, highlighting the preliminary nature of this model. We believe that combining genetic features and more detailed phenotypic information in larger preterm cohorts or general population-based samples is a key future direction and may help explain further why some preterm children develop ASD while others do not.

## Conclusions

In conclusion, we demonstrate that ASD genetic liability is similar in ASD-term and ASD-preterm, suggesting that even within preterm, genetic factors play an important role in etiology. Our study did not find evidence of a link between genetic factors and preterm birth in ASD. However, our findings suggest that preterm birth would exacerbate the severity of outcomes in ASD individuals, and this difference may be driven more by environmental factors. As we observed some differences in the rate of ES DNV in preterm individuals compared between ASD and non-ASD, we only suggest that genetic factors may increase the likelihood of a preterm child getting an ASD diagnosis and the diagnosis is not modified by the interaction between multimorbidity and DNV burden. Through the development of our ML model, we demonstrate that integrating phenotype and genetic information is feasible and holds promise for the early prediction of ASD in preterm children at birth. Our study provides insights into the phenotypic characteristics of ASD preterm individuals. We suggest that health screening for preterm birth infants should incorporate the collection of genetic data, as it better supports early clinical identification of ASD and can aid in the guidance of early intervention strategies.

## Supplementary Information

Additional file 1: Supplementary Figs S1–S12 and Supplementary Tables S1–S16.

Additional file 2: Neurodevelopmental disorder-related gene list used in this study.

## Data Availability

Phenotypic and genetic data for the SPARK and SSC cohorts can be accessed by applying at SFARI ([https://base.sfari.org] (https:/base.sfari.org)) [34]. The major scripts and code used to perform the main analysis are publicly available on Zenodo (10.5281/zenodo.16942094) [80]. Additionally, statistics from the main analyses and gene list used in this study have been provided in Supplementary materials to ensure reproducibility and transparency.

## Abbreviations

ASD: Autism spectrum disorder
SPARK: Simons Foundation Powering Autism Research for Knowledge
SSC: Simons Simplex Collection
GS: Genome sequencing
ES: Exome sequencing
GEE: Generalized Estimating Equations
PRS: Polygenic Risk Score
DNV: De novo variants
CNV: Copy number variation
GWAS: Genome-Wide Association Studies
NDD genes: Neurodevelopmental disorder-related genes
ML: Machine learning
CBCL: Child Behavior Checklist
DCDQ: Developmental Coordination Disorder Questionnaire
RBS-R: Repetitive Behavior Scale-Revised
SCQ: Social Communication Questionnaire
Fsiq: Full-Scale Intelligence Quotient
AB: Allele balance
LOF: Loss-of-function
CADD: Combined Annotation Dependent Depletion
PCA: Principal component analysis
PC: Principal components
CI: Confidence interval
RFE: Recursive Feature Elimination
XGBoost: Extreme Gradient Boosting
RF: Random forest
SVM: Linear Support Vector Machine
AUROC: Area Under the Receiver Operating Characteristic Curve
SHAP: SHapley Additive exPlanations
GA: Gestational age at birth
OR: Odds ratio
FDR: False discovery rate

## References

[1] Rosen NE, Lord C, Volkmar FR. The diagnosis of autism: from Kanner to DSM-III to DSM-5 and beyond. J Autism Dev Disord. 2021;51:4253–70. 33624215 10.1007/s10803-021-04904-1 PMC8531066

[2] Lord C, Brugha TS, Charman T, Cusack J, Dumas G, Frazier T, et al. Autism spectrum disorder. Nat Rev Dis Primers. 2020;6:5. 31949163 10.1038/s41572-019-0138-4 PMC8900942

[3] Carlsson T, Molander F, Taylor MJ, Jonsson U, Bölte S. Early environmental risk factors for neurodevelopmental disorders – a systematic review of twin and sibling studies. Dev Psychopathol. 2021;33:1448–95. 32703331 10.1017/S0954579420000620 PMC8564717

[4] Havdahl A, Niarchou M, Starnawska A, Uddin M, van der Merwe C, Warrier V. Genetic contributions to autism spectrum disorder. Psychol Med. 2021;51:2260–73. 33634770 10.1017/S0033291721000192 PMC8477228

[5] Ruzzo EK, Pérez-Cano L, Jung J-Y, Wang L-K, Kashef-Haghighi D, Hartl C, et al. Inherited and de novo genetic risk for autism impacts shared networks. Cell. 2019;178:850-866.e26. 31398340 10.1016/j.cell.2019.07.015 PMC7102900

[6] Bai D, Yip BHK, Windham GC, Sourander A, Francis R, Yoffe R, et al. Association of genetic and environmental factors with autism in a 5-country cohort. JAMA Psychiatr. 2019;76:1035. 10.1001/jamapsychiatry.2019.1411 PMC6646998 31314057

[7] Iossifov I, O’Roak BJ, Sanders SJ, Ronemus M, Krumm N, Levy D, et al. The contribution of de novo coding mutations to autism spectrum disorder. Nature. 2014;515:216–21. 25363768 10.1038/nature13908 PMC4313871

[8] Zhou X, Feliciano P, Shu C, Wang T, Astrovskaya I, Hall JB, et al. Integrating de novo and inherited variants in 42,607 autism cases identifies mutations in new moderate-risk genes. Nat Genet. 2022;54:1305–19. 35982159 10.1038/s41588-022-01148-2 PMC9470534

[9] Wilfert AB, Turner TN, Murali SC, Hsieh P, Sulovari A, Wang T, et al. Recent ultra-rare inherited variants implicate new autism candidate risk genes. Nat Genet. 2021;53:1125–34. 34312540 10.1038/s41588-021-00899-8 PMC8459613

[10] Tammimies K, Marshall CR, Walker S, Kaur G, Thiruvahindrapuram B, Lionel AC, et al. Molecular diagnostic yield of chromosomal microarray analysis and whole-exome sequencing in children with autism spectrum disorder. JAMA. 2015;314:895. 26325558 10.1001/jama.2015.10078

[11] Grove J, Ripke S, Als TD, Mattheisen M, Walters RK, Won H, et al. Identification of common genetic risk variants for autism spectrum disorder. Nat Genet. 2019;51:431–44. 30804558 10.1038/s41588-019-0344-8 PMC6454898

[12] Loughnan RJ, Palmer CE, Makowski C, Thompson WK, Barch DM, Jernigan TL, et al. Unique prediction of developmental psychopathology from genetic and familial risk. Child Psychology Psychiatry. 2022;63:1631–43. 10.1111/jcpp.13649 PMC9691563 35764363

[13] Antaki D, Guevara J, Maihofer AX, Klein M, Gujral M, Grove J, et al. A phenotypic spectrum of autism is attributable to the combined effects of rare variants, polygenic risk and sex. Nat Genet. 2022;54:1284–92. 35654974 10.1038/s41588-022-01064-5 PMC9474668

[14] Crump C, Sundquist J, Sundquist K. Preterm or early term birth and risk of autism. Pediatrics. 2021;148:e2020032300. 34380775 10.1542/peds.2020-032300 PMC9809198

[15] Persson M, Opdahl S, Risnes K, Gross R, Kajantie E, Reichenberg A, et al. Gestational age and the risk of autism spectrum disorder in Sweden, Finland, and Norway: A cohort study. Fassett MJ, editor. PLoS Med. 2020;17:e1003207. 10.1371/journal.pmed.1003207 PMC7508401 32960896

[16] Svensson AC, Sandin S, Cnattingius S, Reilly M, Pawitan Y, Hultman CM, et al. Maternal effects for preterm birth: a genetic epidemiologic study of 630,000 families. Am J Epidemiol. 2009;170:1365–72. 19854802 10.1093/aje/kwp328

[17] Cullen H, Selzam S, Dimitrakopoulou K, Plomin R, Edwards AD. Greater genetic risk for adult psychiatric diseases increases vulnerability to adverse outcome after preterm birth. Sci Rep. 2021;11:11443. 34075065 10.1038/s41598-021-90045-5 PMC8169748

[18] Heikkilä K, Metsälä J, Pulakka A, Nilsen SM, Kivimäki M, Risnes K, et al. Preterm birth and the risk of multimorbidity in adolescence: a multiregister-based cohort study. The Lancet Public Health. 2023;8:e680–90. 37633677 10.1016/S2468-2667(23)00145-7

[19] Allotey J, Zamora J, Cheong-See F, Kalidindi M, Arroyo-Manzano D, Asztalos E, et al. Cognitive, motor, behavioural and academic performances of children born preterm: a meta-analysis and systematic review involving 64 061 children. BJOG. 2018;125:16–25. 29024294 10.1111/1471-0528.14832

[20] Johnson S, Marlow N. Preterm birth and childhood psychiatric disorders. Pediatr Res. 2011;69:11R-18R. 21289534 10.1203/PDR.0b013e318212faa0

[21] Joseph RM, Lai ER, Bishop S, Yi J, Bauman ML, Frazier JA, et al. Comparing autism phenotypes in children born extremely preterm and born at term. Autism Res. 2023;16:653–66. 36595641 10.1002/aur.2885 PMC10551822

[22] Leoni M, Vanes LD, Hadaya L, Kanel D, Dazzan P, Simonoff E, et al. Exploring cognitive, behavioral and autistic trait network topology in very preterm and term-born children. Front Psychol. 2023;14:1119196. 37187563 10.3389/fpsyg.2023.1119196 PMC10176608

[23] Luu J, Jellett R, Yaari M, Gilbert M, Barbaro J. A comparison of children born preterm and full-term on the autism spectrum in a prospective community sample. Front Neurol. 2020;11:597505. 33343497 10.3389/fneur.2020.597505 PMC7744721

[24] Li J, Oehlert J, Snyder M, Stevenson DK, Shaw GM. Fetal de novo mutations and preterm birth. Williams SM, editor. PLoS Genet. 2017;13:e1006689. 10.1371/journal.pgen.1006689 PMC5384656 28388617

[25] Wong HS, Wadon M, Evans A, Kirov G, Modi N, O’Donovan MC, et al. Contribution of de novo and inherited rare CNVs to very preterm birth. J Med Genet. 2020;57:552–7. 32051258 10.1136/jmedgenet-2019-106619

[26] Everett SS, Bomback M, Sahni R, Wapner RJ, Tolia VN, Clark RH, et al. Prevalence and clinical significance of commonly diagnosed genetic disorders in preterm infants. 2023 . Available from: http://medrxiv.org/lookup/doi/10.1101/2023.07.14.23292662. Cited 2024 Sep 20.

[27] Chen J, Engelhard M, Henao R, Berchuck S, Eichner B, Perrin EM, et al. Enhancing early autism prediction based on electronic records using clinical narratives. J Biomed Inform. 2023;144:104390. 37182592 10.1016/j.jbi.2023.104390 PMC10526711

[28] Rajagopalan SS, Zhang Y, Yahia A, Tammimies K. Machine learning prediction of autism spectrum disorder from a minimal set of medical and background information. JAMA Netw Open. 2024;7:e2429229. 39158907 10.1001/jamanetworkopen.2024.29229 PMC11333987

[29] Asif M, Martiniano HFMC, Marques AR, Santos JX, Vilela J, Rasga C, et al. Identification of biological mechanisms underlying a multidimensional ASD phenotype using machine learning. Transl Psychiatry. 2020;10:43. 32066720 10.1038/s41398-020-0721-1 PMC7026098

[30] Li D, Choque Olsson N, Becker M, Arora A, Jiao H, Norgren N, et al. Rare variants in the outcome of social skills group training for autism. Autism Res. 2022;15:434–46. 34968013 10.1002/aur.2666

[31] Bowe AK, Lightbody G, Staines A, Murray DM, Norman M. Prediction of 2-year cognitive outcomes in very preterm infants using machine learning methods. JAMA Netw Open. 2023;6:e2349111. 38147334 10.1001/jamanetworkopen.2023.49111 PMC10751596

[32] Feliciano P, Daniels AM, Green Snyder L, Beaumont A, Camba A, Esler A, et al. SPARK: a US cohort of 50,000 families to accelerate autism research. Neuron. 2018;97:488–93. 29420931 10.1016/j.neuron.2018.01.015 PMC7444276

[33] Fischbach GD, Lord C. The simons simplex collection: a resource for identification of autism genetic risk factors. Neuron. 2010;68:192–5. 20955926 10.1016/j.neuron.2010.10.006

[34] Simons Foundation Autism Research Initiative (SFARI). 2025. Available from: https://www.sfari.org/. Cited 2025 Aug 18.

[35] Ng JK, Vats P, Fritz-Waters E, Sarkar S, Sams EI, Padhi EM, et al. De novo variant calling identifies cancer mutation signatures in the 1000 genomes project. Hum Mutat. 2022;43:1979–93. 36054329 10.1002/humu.24455 PMC9771978

[36] Weiner DJ, Wigdor EM, Ripke S, Walters RK, Kosmicki JA, Grove J, et al. Polygenic transmission disequilibrium confirms that common and rare variation act additively to create risk for autism spectrum disorders. Nat Genet. 2017;49:978–85. 28504703 10.1038/ng.3863 PMC5552240

[37] Achenbach TM. Manual for ASEBA school-age forms & profiles. Youth & Families: University of Vermont, Research Center for Children; 2001.

[38] Wilson BN, Crawford SG, Green D, Roberts G, Aylott A, Kaplan BJ. Psychometric properties of the revised developmental coordination disorder questionnaire. Phys Occup Ther Pediatr. 2009;29:182–202. 19401931 10.1080/01942630902784761

[39] Lam KSL, Aman MG. The repetitive behavior scale-revised: independent validation in individuals with autism spectrum disorders. J Autism Dev Disord. 2007;37:855–66. 17048092 10.1007/s10803-006-0213-z

[40] Rutter M, Bailey A, Lord C. The social communication questionnaire. Los Angeles, CA: Western Psychological Services. 2003.

[41] Wechsler D. Wechsler intelligence scale for children–Fourth Edition (WISC-IV). San Antonio: Harcourt Assessment. 2003.

[42] Pedersen BS, Brown JM, Dashnow H, Wallace AD, Velinder M, Tristani-Firouzi M, et al. Effective variant filtering and expected candidate variant yield in studies of rare human disease. NPJ Genom Med. 2021;6:60. 34267211 10.1038/s41525-021-00227-3 PMC8282602

[43] McKenna A, Hanna M, Banks E, Sivachenko A, Cibulskis K, Kernytsky A, et al. The genome analysis toolkit: a mapreduce framework for analyzing next-generation DNA sequencing data. Genome Res. 2010;20:1297–303. 20644199 10.1101/gr.107524.110 PMC2928508

[44] Wang K, Li M, Hakonarson H. ANNOVAR: functional annotation of genetic variants from high-throughput sequencing data. Nucleic Acids Res. 2010;38:e164–e164. 20601685 10.1093/nar/gkq603 PMC2938201

[45] Cingolani P, Platts A, Wang LL, Coon M, Nguyen T, Wang L, et al. A program for annotating and predicting the effects of single nucleotide polymorphisms, SnpEff: SNPs in the genome of *Drosophila melanogaster* strain w 1118; iso-2; iso-3. Fly. 2012;6:80–92. 22728672 10.4161/fly.19695 PMC3679285

[46] McLaren W, Gil L, Hunt SE, Riat HS, Ritchie GRS, Thormann A, et al. The ensembl variant effect predictor. Genome Biol. 2016;17:122. 27268795 10.1186/s13059-016-0974-4 PMC4893825

[47] Abrahams BS, Arking DE, Campbell DB, Mefford HC, Morrow EM, Weiss LA, et al. SFARI gene 2.0: a community-driven knowledgebase for the autism spectrum disorders (ASDs). Mol Autism. 2013;4:36. 24090431 10.1186/2040-2392-4-36 PMC3851189

[48] Martin AR, Williams E, Foulger RE, Leigh S, Daugherty LC, Niblock O, et al. Panelapp crowdsources expert knowledge to establish consensus diagnostic gene panels. Nat Genet. 2019;51:1560–5. 31676867 10.1038/s41588-019-0528-2

[49] Danecek P, Auton A, Abecasis G, Albers CA, Banks E, DePristo MA, et al. The variant call format and VCFtools. Bioinformatics. 2011;27:2156–8. 21653522 10.1093/bioinformatics/btr330 PMC3137218

[50] Kochinke K, Zweier C, Nijhof B, Fenckova M, Cizek P, Honti F, et al. Systematic phenomics analysis deconvolutes genes mutated in intellectual disability into biologically coherent modules. Am J Hum Genet. 2016;98:149–64. 26748517 10.1016/j.ajhg.2015.11.024 PMC4716705

[51] Firth HV, Richards SM, Bevan AP, Clayton S, Corpas M, Rajan D, et al. DECIPHER: Database of chromosomal imbalance and phenotype in humans using Ensembl resources. Am J Hum Genet. 2009;84:524–33. 19344873 10.1016/j.ajhg.2009.03.010 PMC2667985

[52] Chang CC, Chow CC, Tellier LC, Vattikuti S, Purcell SM, Lee JJ. Second-generation PLINK: rising to the challenge of larger and richer datasets. Gigascience. 2015;4:7. 25722852 10.1186/s13742-015-0047-8 PMC4342193

[53] Hinrichs AS. The UCSC Genome Browser Database: update 2006. Nucleic Acids Res. 2006;34:D590–8. 16381938 10.1093/nar/gkj144 PMC1347506

[54] Ge T, Chen C-Y, Ni Y, Feng Y-CA, Smoller JW. Polygenic prediction via Bayesian regression and continuous shrinkage priors. Nat Commun. 2019;10:1776. 30992449 10.1038/s41467-019-09718-5 PMC6467998

[55] Halekoh U, Højsgaard S, Yan J. The R package geepack for generalized estimating equations. J Stat Soft. 2006;15. Available from: http://www.jstatsoft.org/v15/i02/. Cited 2024 May 7.

[56] Lüdecke D. Ggeffects: tidy data frames of marginal effects from regression models. J Open Source Softw. 2018;3:772.

[57] Gregorutti B, Michel B, Saint-Pierre P. Correlation and variable importance in random forests. Stat Comput. 2017;27:659–78.

[58] Kuhn M. Building predictive models in R using the caret package. J Stat Soft. 2008;28:1–26.

[59] Liu Y, Just A. SHAPforxgboost: SHAP Plots for “XGBoost”. 2020. Available from: https://github.com/liuyanguu/SHAPforxgboost/

[60] Heikkilä K, Pulakka A, Metsälä J, Alenius S, Hovi P, Gissler M, et al. Preterm birth and the risk of chronic disease multimorbidity in adolescence and early adulthood: a population-based cohort study. Jacobsen R, editor. PLoS ONE. 2021;16:e0261952. 10.1371/journal.pone.0261952 PMC8719774 34972182

[61] Johnson S, Marlow N. Growing up after extremely preterm birth: lifespan mental health outcomes. Semin Fetal Neonatal Med. 2014;19:97–104. 24290907 10.1016/j.siny.2013.11.004

[62] Hirvonen M, Ojala R, Korhonen P, Haataja P, Eriksson K, Gissler M, et al. Visual and hearing impairments after preterm birth. Pediatrics. 2018;142:e20173888. 30018154 10.1542/peds.2017-3888

[63] Li W, Peng A, Deng S, Lai W, Qiu X, Zhang L, et al. Do premature and postterm birth increase the risk of epilepsy? An updated meta-analysis. Epilepsy Behav. 2019;97:83–91. 31202097 10.1016/j.yebeh.2019.05.016

[64] Khachadourian V, Mahjani B, Sandin S, Kolevzon A, Buxbaum JD, Reichenberg A, et al. Comorbidities in autism spectrum disorder and their etiologies. Transl Psychiatry. 2023;13:71. 36841830 10.1038/s41398-023-02374-w PMC9958310

[65] Johnson S, Hollis C, Kochhar P, Hennessy E, Wolke D, Marlow N. Autism spectrum disorders in extremely preterm children. J Pediatr. 2010;156:525-531.e2. 20056232 10.1016/j.jpeds.2009.10.041

[66] Schieve LA, Baio J, Rice CE, Durkin M, Kirby RS, Drews-Botsch C, et al. Risk for cognitive deficit in a population-based sample of U.S. children with autism spectrum disorders: variation by perinatal health factors. Disabil Health J. 2010;3:202–12. 21122785 10.1016/j.dhjo.2009.12.001

[67] Zhu JL, Olsen J, Olesen AW. Risk for developmental coordination disorder correlates with gestational age at birth. Paediatr Perinat Epidemiol. 2012;26:572–7. 23061693 10.1111/j.1365-3016.2012.01316.x

[68] York TP, Eaves LJ, Lichtenstein P, Neale MC, Svensson A, Latendresse S, et al. Fetal and maternal genes’ influence on gestational age in a quantitative genetic analysis of 244,000 Swedish births. Am J Epidemiol. 2013;178:543–50. 23568591 10.1093/aje/kwt005 PMC3736752

[69] Li C, Fleck JS, Martins-Costa C, Burkard TR, Themann J, Stuempflen M, et al. Single-cell brain organoid screening identifies developmental defects in autism. Nature. 2023;621:373–80. 37704762 10.1038/s41586-023-06473-y PMC10499611

[70] Solé-Navais P, Flatley C, Steinthorsdottir V, Vaudel M, Juodakis J, Chen J, et al. Genetic effects on the timing of parturition and links to fetal birth weight. Nat Genet. 2023;55:559–67. 37012456 10.1038/s41588-023-01343-9 PMC10101852

[71] Liu X, Helenius D, Skotte L, Beaumont RN, Wielscher M, Geller F, et al. Variants in the fetal genome near pro-inflammatory cytokine genes on 2q13 associate with gestational duration. Nat Commun. 2019;10:3927. 31477735 10.1038/s41467-019-11881-8 PMC6718389

[72] Zhang G, Feenstra B, Bacelis J, Liu X, Muglia LM, Juodakis J, et al. Genetic associations with gestational duration and spontaneous preterm birth. N Engl J Med. 2017;377:1156–67. 28877031 10.1056/NEJMoa1612665 PMC5561422

[73] Eklöf E, Mårtensson GE, Ådén U, Padilla N. Reduced structural brain asymmetry during neonatal life is potentially related to autism spectrum disorders in children born extremely preterm. Autism Res. 2019;12:1334–43. 31287626 10.1002/aur.2169

[74] Padilla N, Eklöf E, Mårtensson GE, Bölte S, Lagercrantz H, Ådén U. Poor brain growth in extremely preterm neonates long before the onset of autism spectrum disorder symptoms. Cerebral Cortex. 2015;27:1245–52. 10.1093/cercor/bhv300 26689588

[75] Hadaya L, Vanes L, Karolis V, Kanel D, Leoni M, Happé F, et al. Distinct neurodevelopmental trajectories in groups of very preterm children screening positively for autism spectrum conditions. J Autism Dev Disord. 2024;54:256–69. 36273367 10.1007/s10803-022-05789-4 PMC10791910

[76] Hee Chung E, Chou J, Brown KA. Neurodevelopmental outcomes of preterm infants: a recent literature review. Transl Pediatr. 2020;9:S3-8. 32206579 10.21037/tp.2019.09.10 PMC7082240

[77] Amit G, Bilu Y, Sudry T, Avgil Tsadok M, Zimmerman DR, Baruch R, et al. Early prediction of autistic spectrum disorder using developmental surveillance data. JAMA Netw Open. 2024;7:e2351052. 38198135 10.1001/jamanetworkopen.2023.51052 PMC12282497

[78] Hyman SL, Levy SE, Myers SM, Council on children with disabilities, section on developmental and behavioral pediatrics, Kuo DZ, Apkon S, et al. Identification, evaluation, and management of children with autism spectrum disorder. Pediatrics. 2020;145:e20193447. 31843864 10.1542/peds.2019-3447

[79] Sajdel-Sulkowska EM, Makowska-Zubrycka M, Czarzasta K, Kasarello K, Aggarwal V, Bialy M, et al. Common genetic variants link the abnormalities in the gut-brain axis in prematurity and autism. Cerebellum. 2019;18:255–65. 30109601 10.1007/s12311-018-0970-1 PMC6443615

[80] Zhang Y. Prematurity and genetic liability for autism spectrum disorder: main analysis and figure code. Zenodo; 2025. Available from: 10.5281/zenodo.16942094 10.1186/s13073-025-01552-3 PMC12490119 41039503

