# Supplementary material for: Prematurity and genetic liability for autism spectrum disorder
Source: Genome Med. 2025 Oct 2;17:108. doi: 10.1186/s13073-025-01552-3 (PMC12490119; doi:10.1186/s13073-025-01552-3)
Supplement: Supplementary file 1 — Additional file 1: Supplementary Figs S1–S12 and Supplementary Tables S1–S16. [file 13073_2025_1552_MOESM1_ESM.pdf]

Additional file 1

Supplementary Figs S1-12

Supplementary Tables S1-16

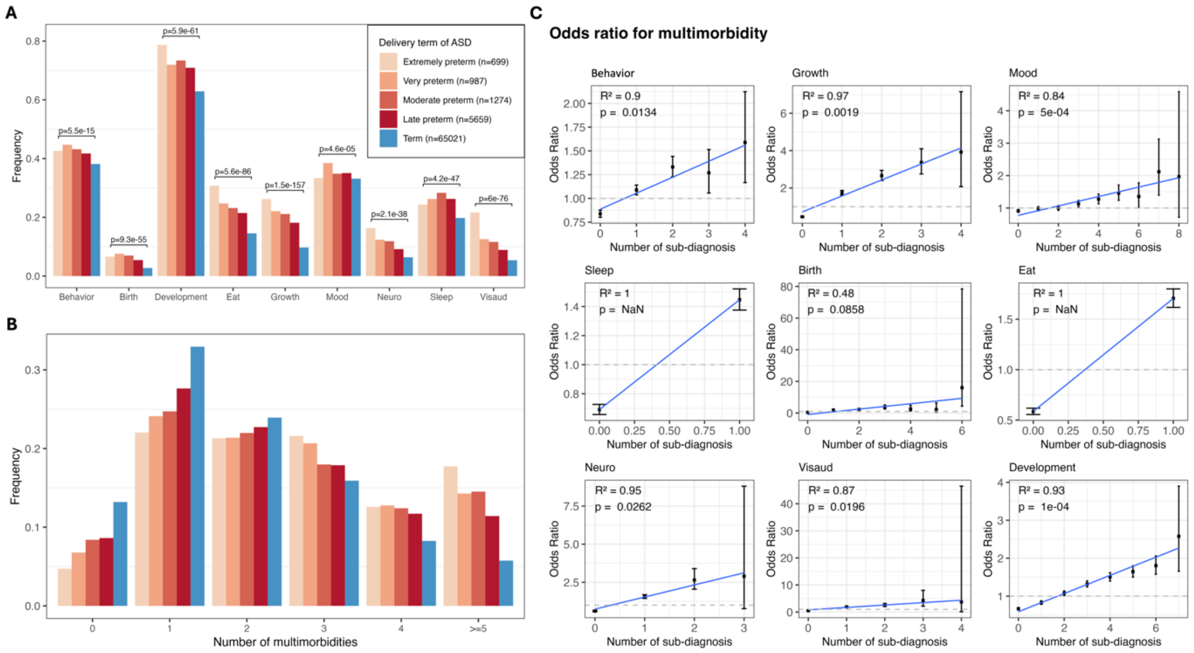

Fig S1. The phenotype comparison between different preterm stages within ASD regardless of genotype (for the SPARK cohort). Color bars are the same across three panels and shown in panel A. A. Prevalence of diagnostic categories. Significance was assessed using χ^2 test with FDR-adjusted p-value (details of diagnostic categories are described in Table S1). B. Distribution of the number of multimorbidities (number of diagnostic categories detailed in Table S1). The bar color and sample size for each group are the same as in A. The significance of differences in prevalence between different preterm stages is recorded in Table S9. C. The odds ratio (OR) with 95% CI for the frequency of the number of multimorbidities in each diagnostic category. ORs are given among ASD individuals born preterm vs term. The Pearson test was used to assess the R^2 and p-value of linear regression.

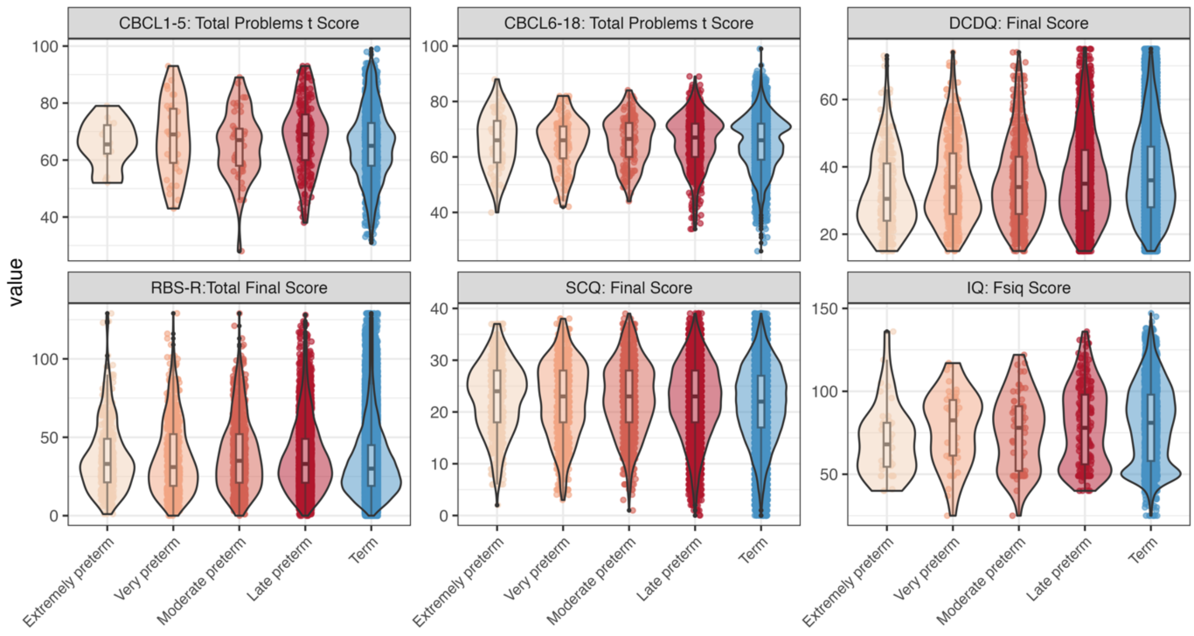

Fig S2. Differences in quantitative measures among ASD individuals in different preterm stages and term (for SPARK cohort). Measures include Child Behavior Checklist (CBCL) t score for 1 to 5 and 6 to 18 years of age, Developmental Coordination Disorder Questionnaire (DCDQ), Repetitive Behavior Scale-Revised (RBS-R) score, Social Communication Questionnaire (SCQ), and Full-Scale IQ (Fsiq). Score distributions of different stages were compared using Kruskal-Wallis rank sum test, except CBCL6-18 (p=0.089), IQ (p=0.066), others have p<0.05. In the pairwise Wilcoxon rank sum test, extremely preterm have lower DCDQ final score than very preterm (p=0.003), moderate preterm (p=0.002), and late preterm ($p=2.7\times10^{-5}$) born, with other pairs having p>0.05.

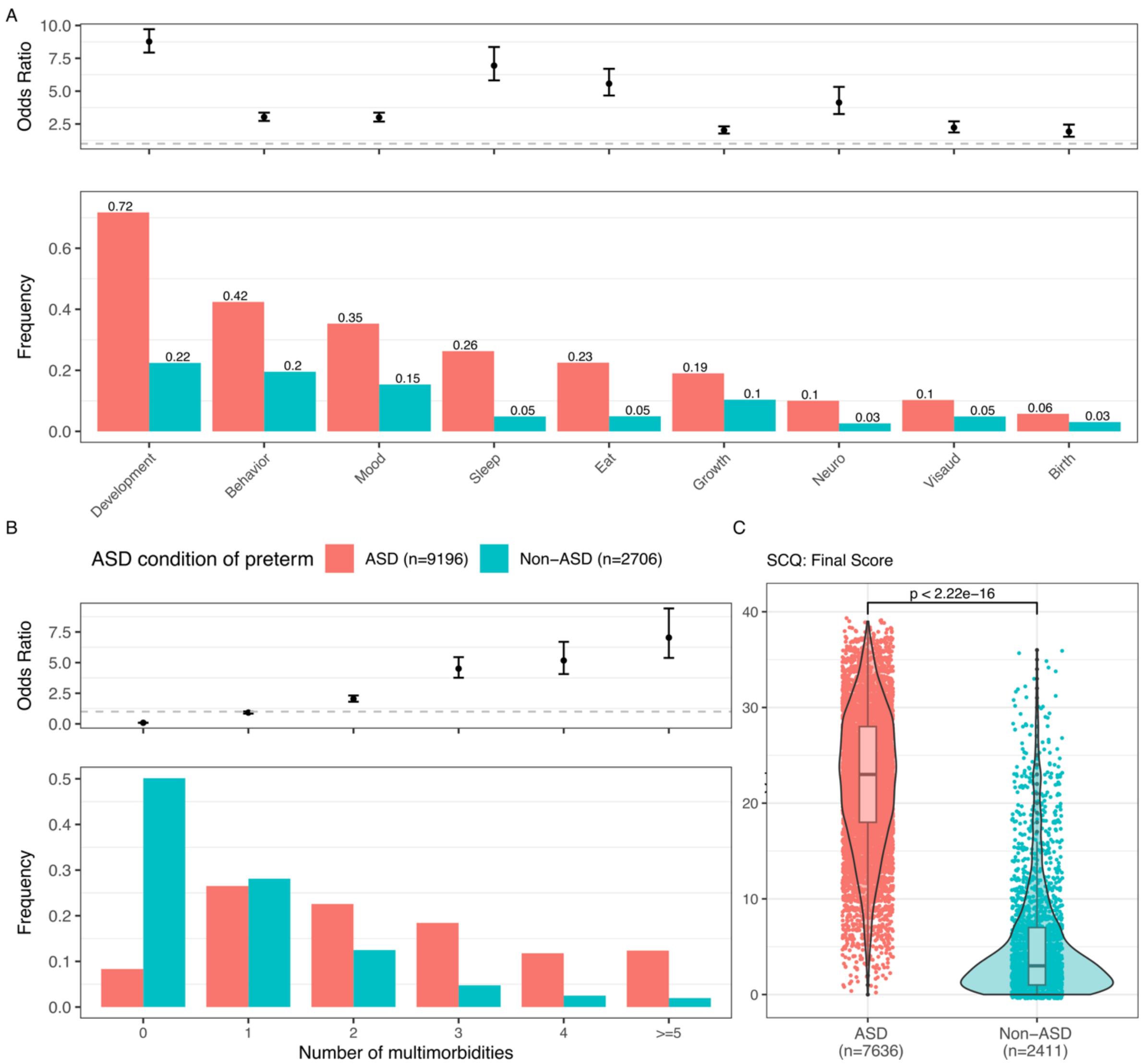

Fig S3. The phenotype comparison between ASD and non-ASD within preterm individuals in SPARK cohort. Color bars are the same across three panels and shown at the top of panel B. A. Prevalence and odds ratio with 95% confidence interval (CI) of diagnostic categories. The detailed outcomes in each diagnostic category are described in Table S1. The exact prevalence values are labeled on the top of the bars. ORs are given among ASD individuals vs non-ASD individuals, recorded in Table S12. B. Distribution of the number of multimorbidities, defined as the total number of diagnostic categories (Table S1) that appeared in individuals. ORs with 95% CI are given among ASD individuals vs non-ASD individuals when focusing on each multimorbidity number and recorded in Table S13. C. Differences in Social Communication Questionnaire (SCQ) among preterm individuals with ASD and without ASD. Significance was assessed using the 2-sided Wilcoxon signed-rank test with the p-value marked in the plot as 0-0.001***.

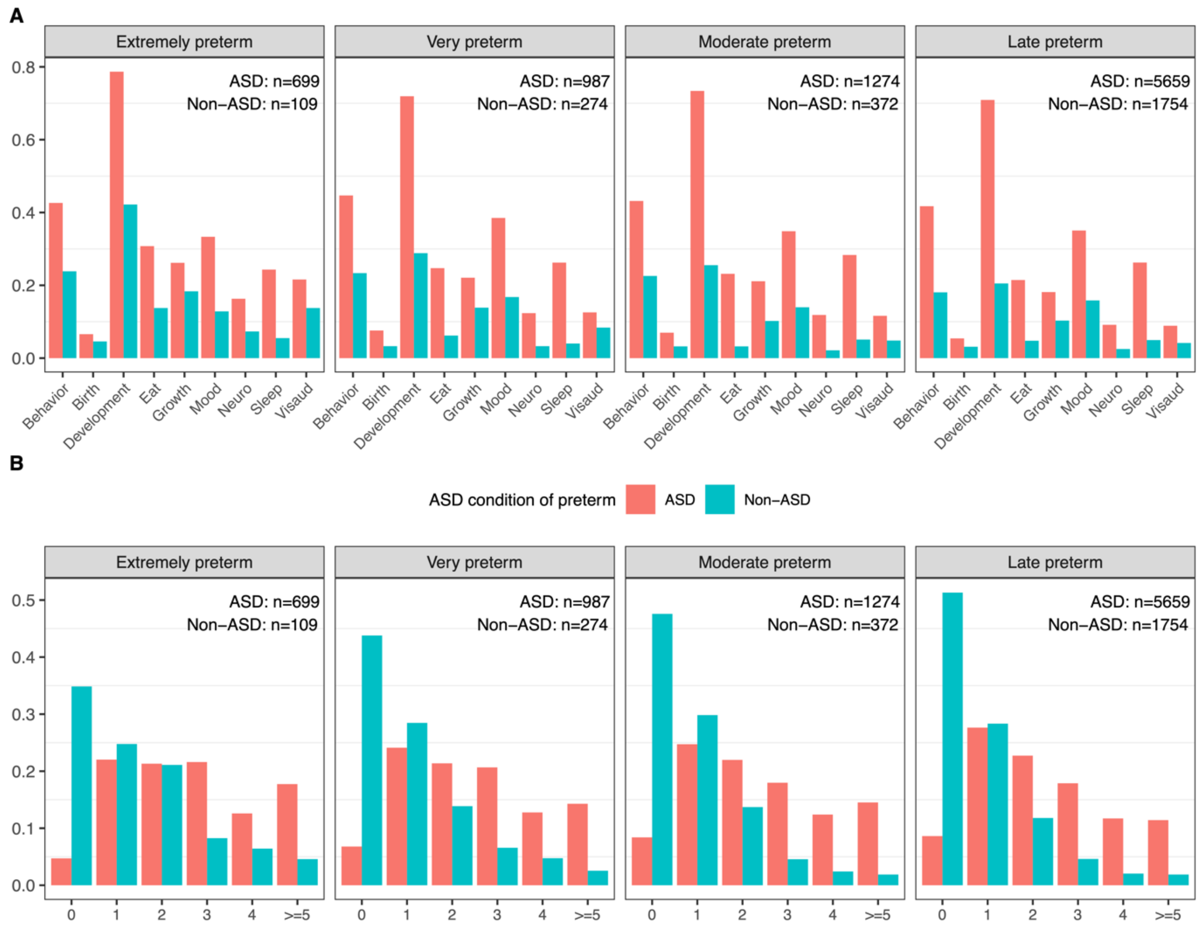

Fig S4. The phenotype comparison between ASD and non-ASD within different preterm stages in SPARK cohort. A. Prevalence of diagnosis. B. Distribution of the number of multimorbidities. The sample size of each subgroup is labeled in each panel.

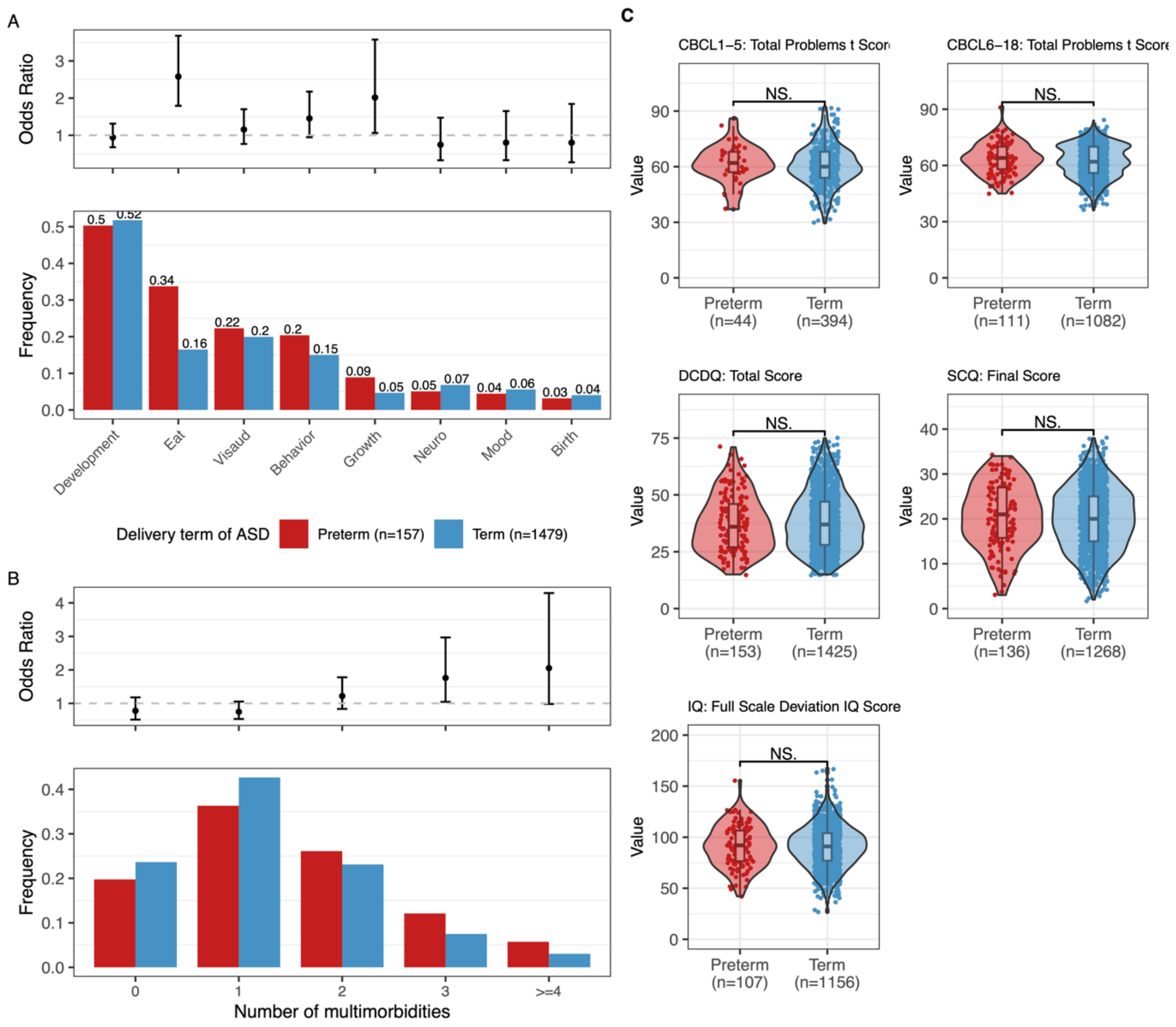

Fig S5. The phenotype comparison between preterm and term within ASD in the SSC cohort. A. Prevalence and odds ratio with 95% confidence interval (CI) of diagnostic categories. The detailed outcomes in each diagnostic category are described in Table S8. The exact prevalence values are labeled on the top of the bars. ORs are given among ASD individuals born preterm vs term and recorded in Table S14. B. Distribution of the number of multimorbidities, defined as the total number of diagnostic categories (Table S8) that appeared in individuals. ORs with 95% CI are given among ASD individuals born preterm vs term when focusing on each multimorbidity number and recorded in Table S15. C. Differences in Child Behavior Checklist (CBCL) t score for 1 to 5 and 6 to 18 years of age, Developmental Coordination Disorder Questionnaire (DCDQ), Social Communication Questionnaire (SCQ), and Full-Scale IQ among ASD individuals born preterm and term. Significance was assessed using the 2-sided Wilcoxon signed-rank test with the p-value marked in the plots as NS (non-significant difference).

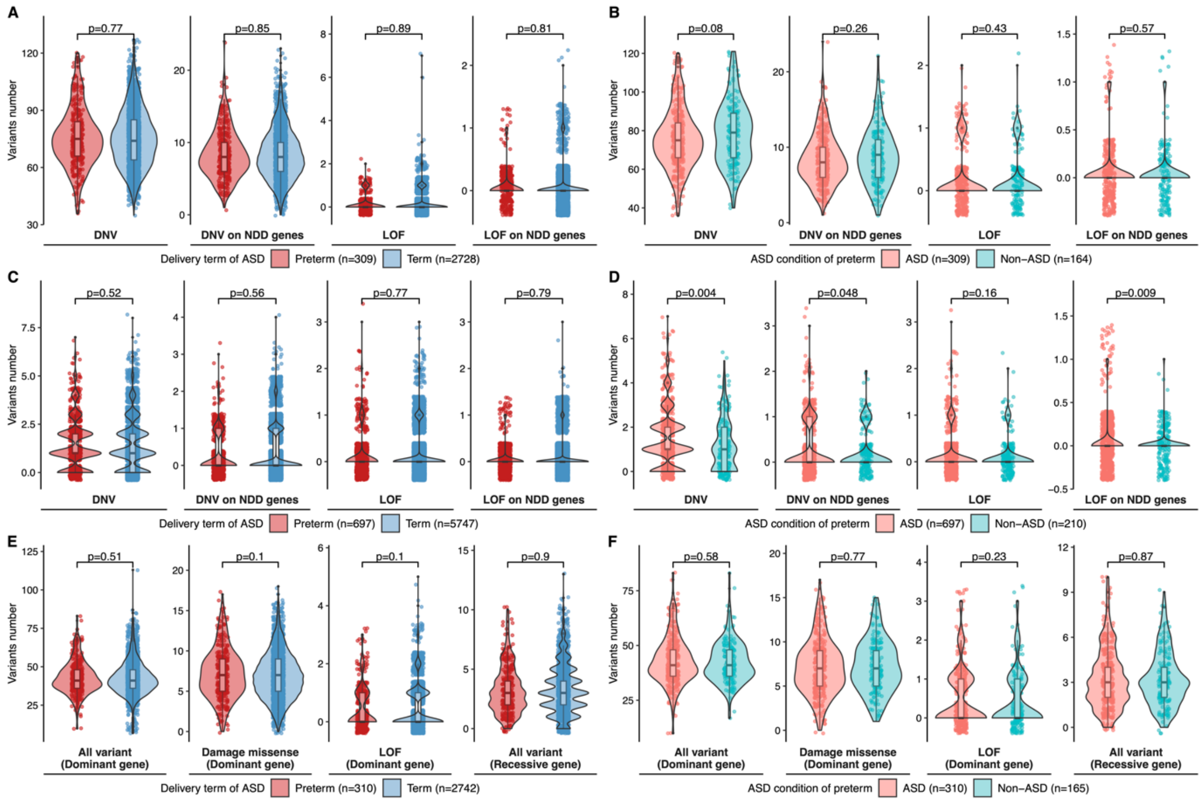

Fig S6. The distribution of genetic variants number in SPARK cohort. In ASD individuals, the number of de novo variants identified through whole genome sequencing (A) and whole exome sequencing (C), and inherited variants on dominant and recessive NDD genes identified through whole genome sequencing (E) for each individual were shown. In preterm individuals, the number of de novo variants identified through whole genome sequencing (B) and whole exome sequencing (D), and inherited variants on dominant and recessive NDD genes identified through whole genome sequencing (F) for each individual were shown. Differences between groups were assessed using the 2-sided Wilcoxon signed-rank test.

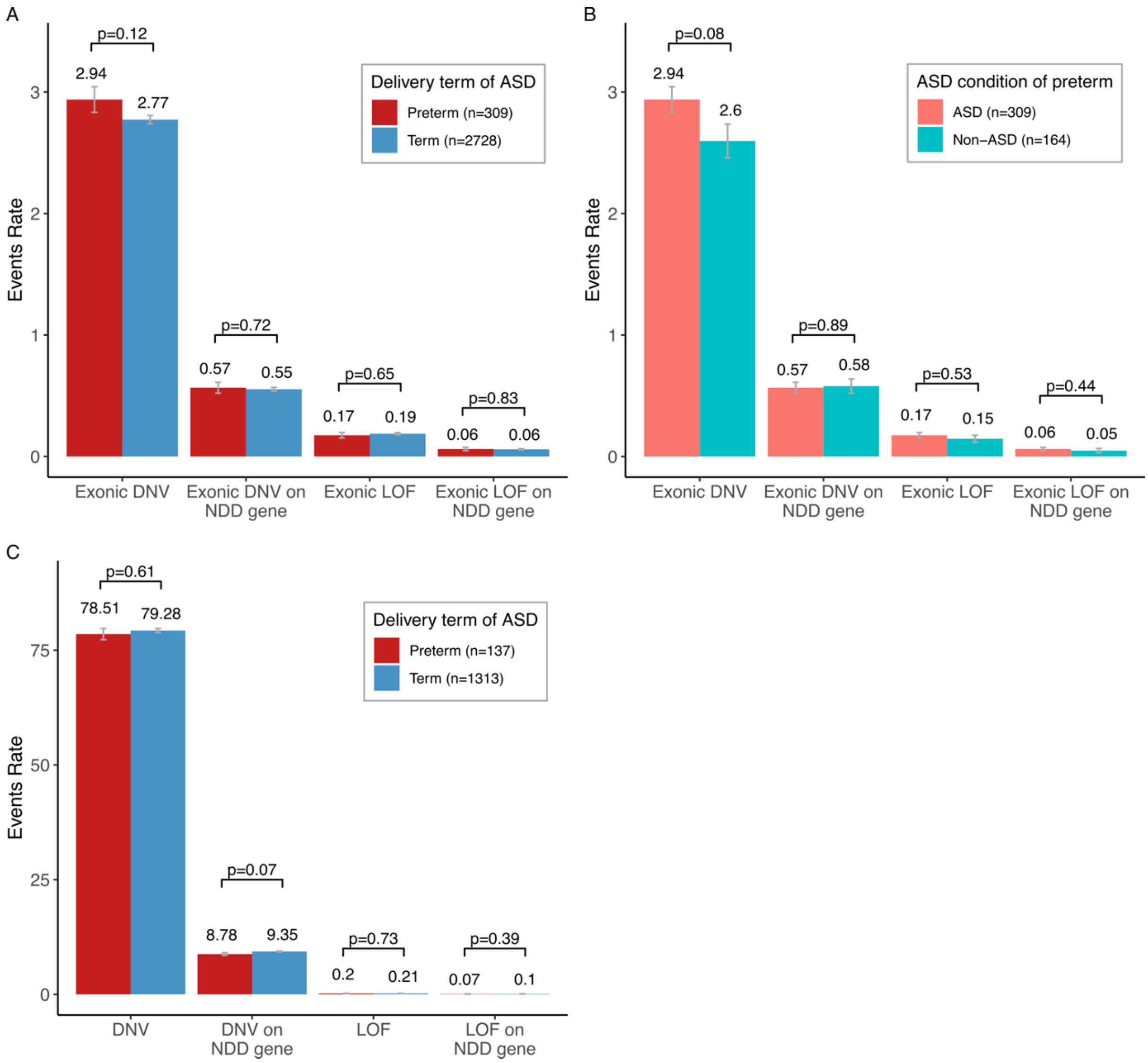

Fig S7. Association between genetic variant burden and subgroups with varying preterm birth and ASD status. In SPARK, for de novo variants found on exonic regions in GS, event rates were compared between ASD-preterm and ASD-term (A), and between ASD-preterm and Non-ASD-preterm (B). C. The event rates of de novo variants were compared between preterm and term within ASD individuals in the SSC cohort. Data are presented as mean values ± standard errors as error bars. GEE model with Poisson family and sex covariate was used to compute the p-value to assess the differences in variant count between groups.

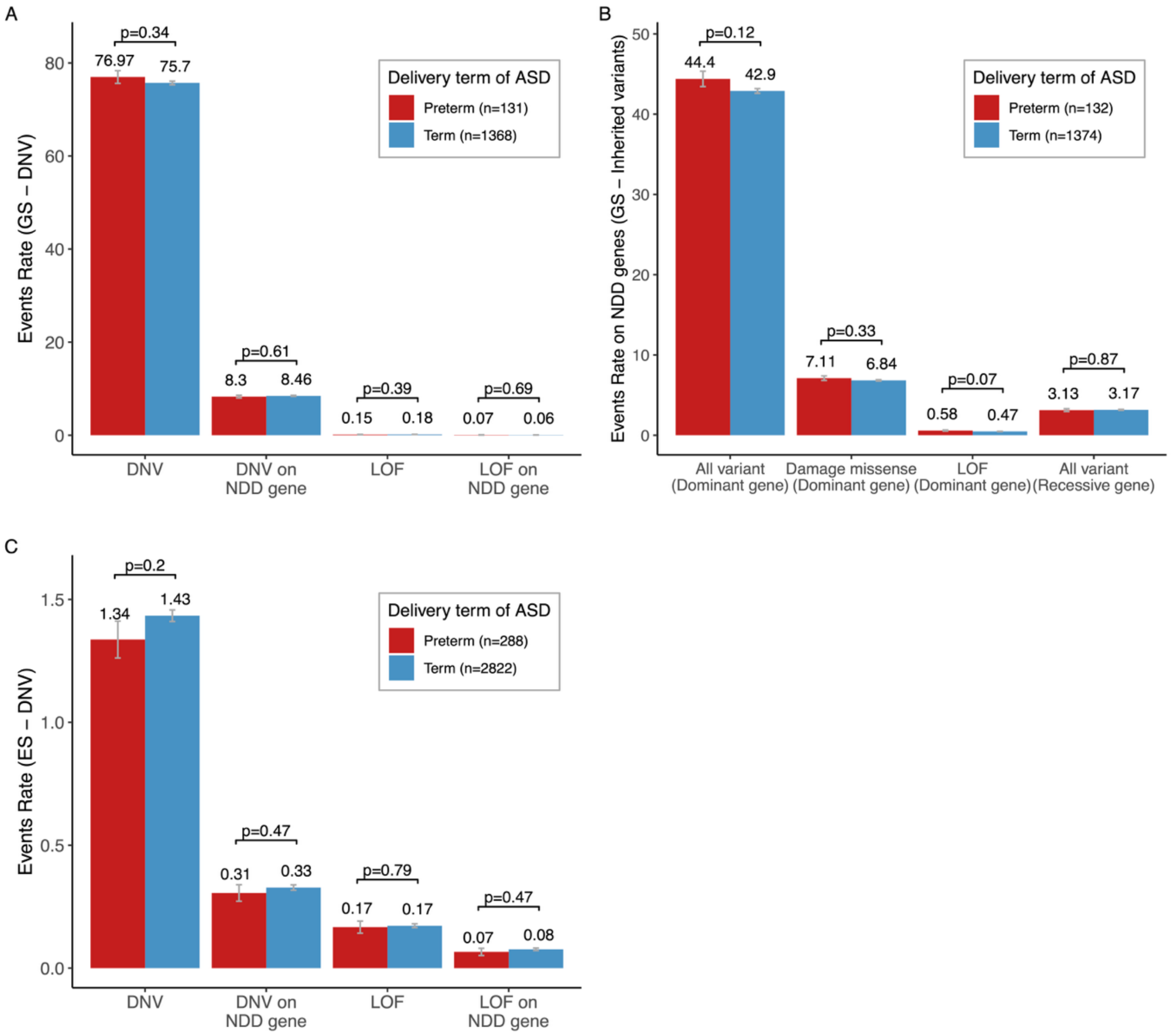

Fig S8. Association between genetic variant burden and subgroups with varying preterm birth status for probands from simplex families in SPARK. Event rates of rare de novo variants (DNV) and inherited variants on dominant and recessive NDD genes (B) identified through genome sequencing, and DNV identified through exome sequencing (C) were calculated. Data are presented as mean values ± standard errors as error bars. GEE model with Poisson family and sex covariate was used to compute the p-value to assess the differences in variant count between groups.

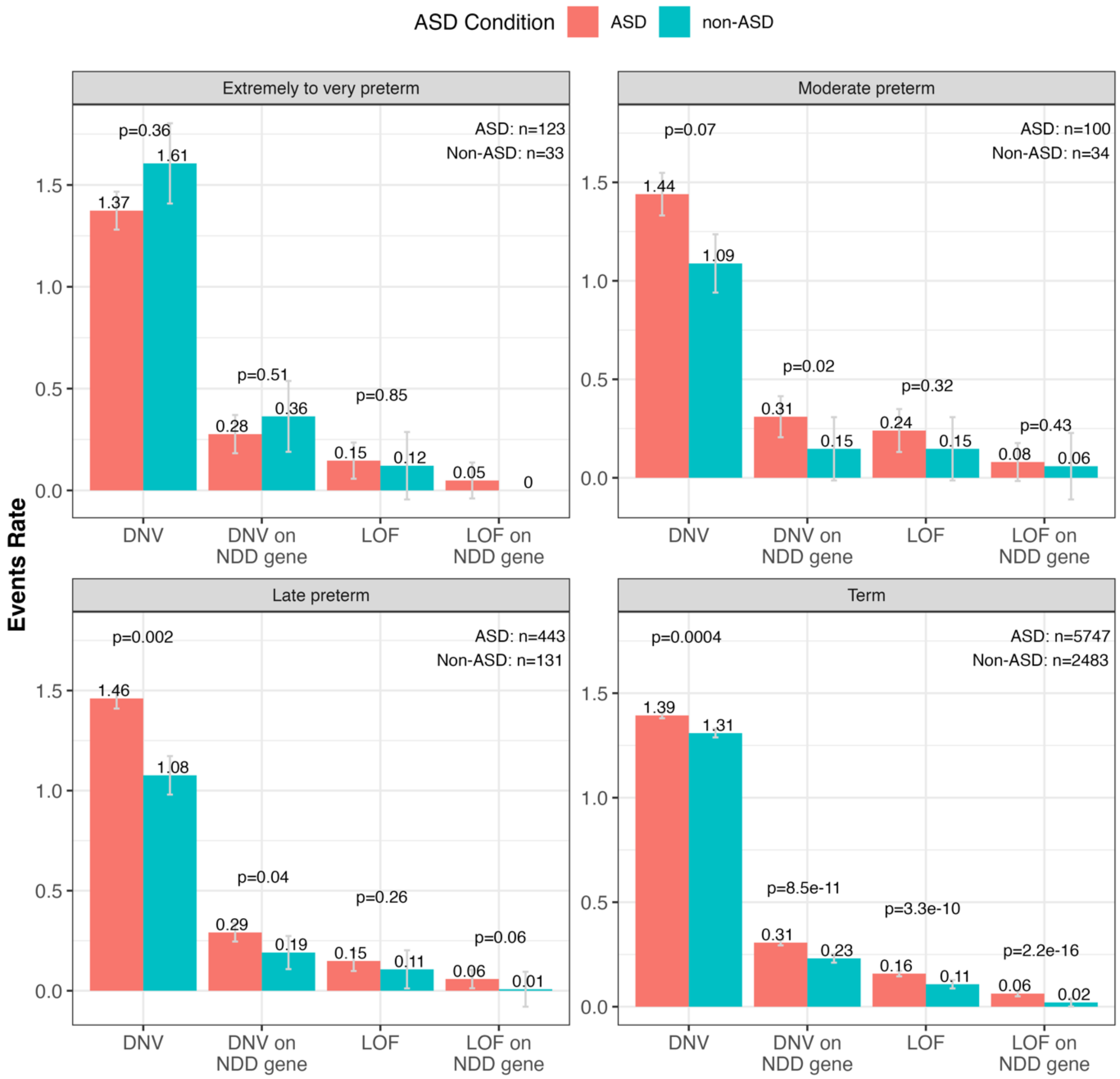

Fig S9. The event rates of de novo variants identified in WES were compared between ASD and non-ASD in SPARK individuals in different preterm stages and term. Data are presented as mean values ± standard errors as error bars. GEE model with Poisson family and sex covariate was used to compute the p-value to assess the differences in variant count between groups.

A

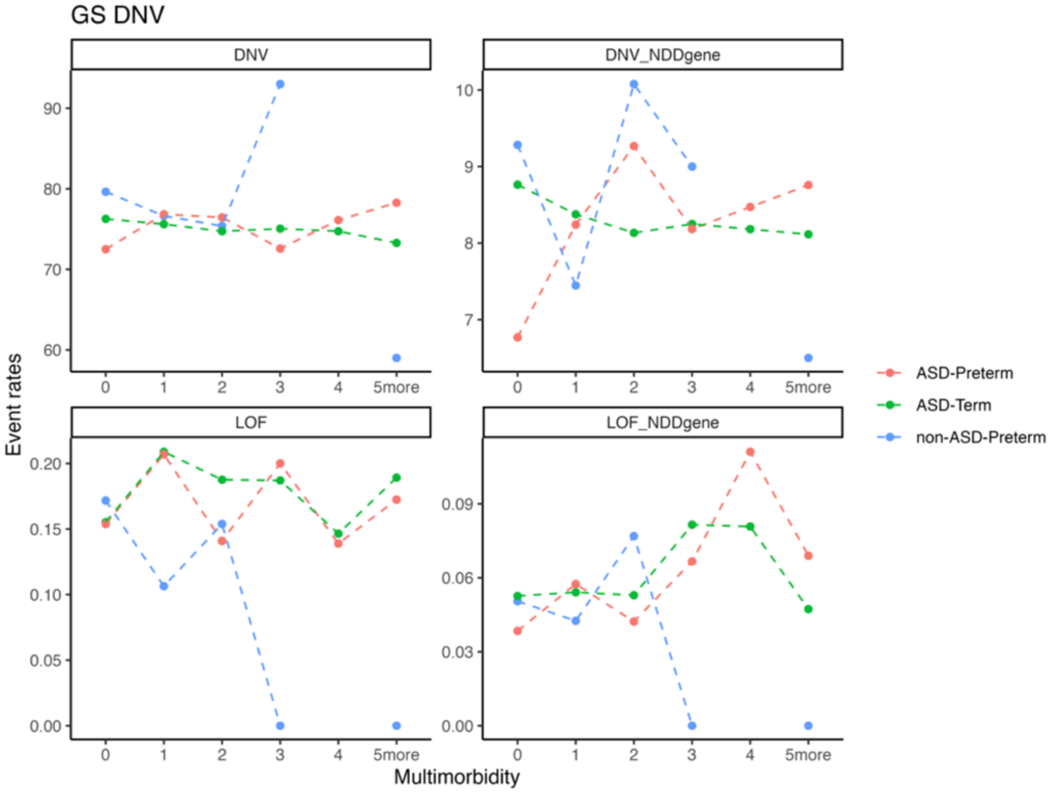

B

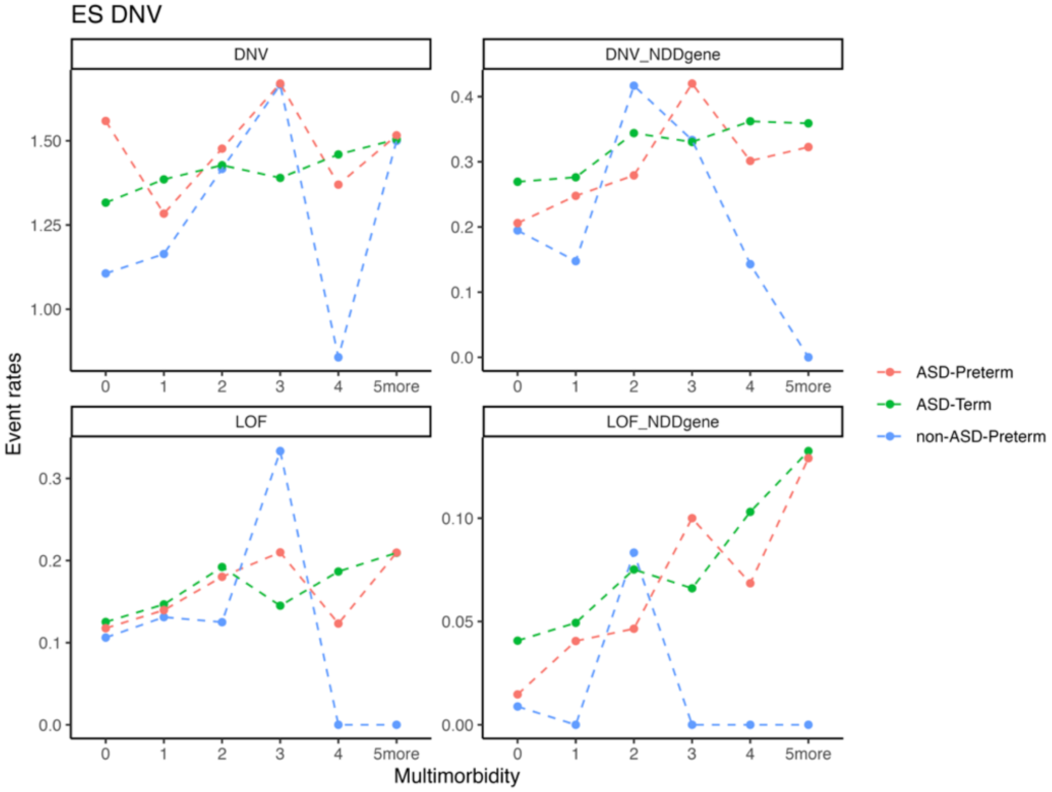

Fig S10. DNV event rates across multimorbidity categories in different ASD and preterm status (SPARK cohort). Variants identified in GS (A) and ES (B) are grouped in DNV, DNV in NDD genes (DNV_NDDgene), loss-of-function (LOF) variants, and LOF variants in NDD genes (LOF_NDDgene).

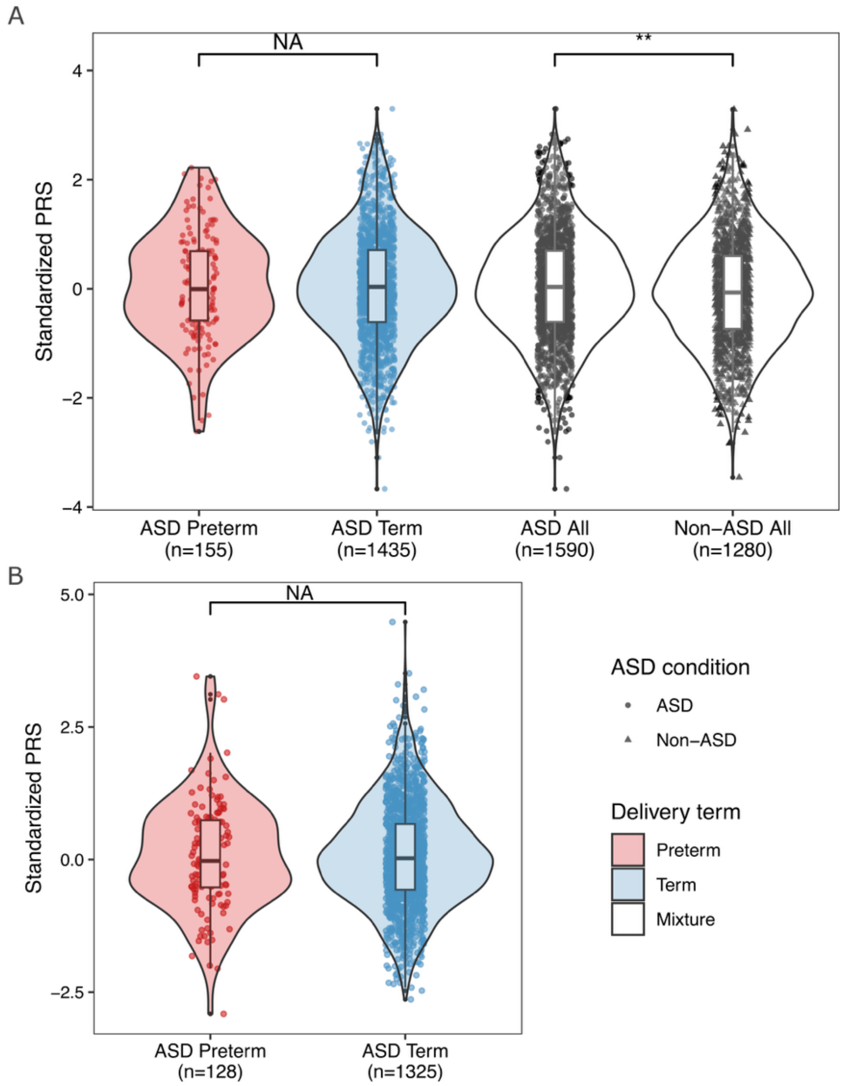

Fig S11. The distribution of standardized PRS in SSC cohort individuals (A) and SPARK simplex family probands (B) with different prematurity and ASD status. Significance was assessed using the 2-sided Wilcoxon signed-rank test with the p-value marked in the plots as 0-0.001***, 0.001-0.01**, 0.01-0.05* or NS (non-significant difference).

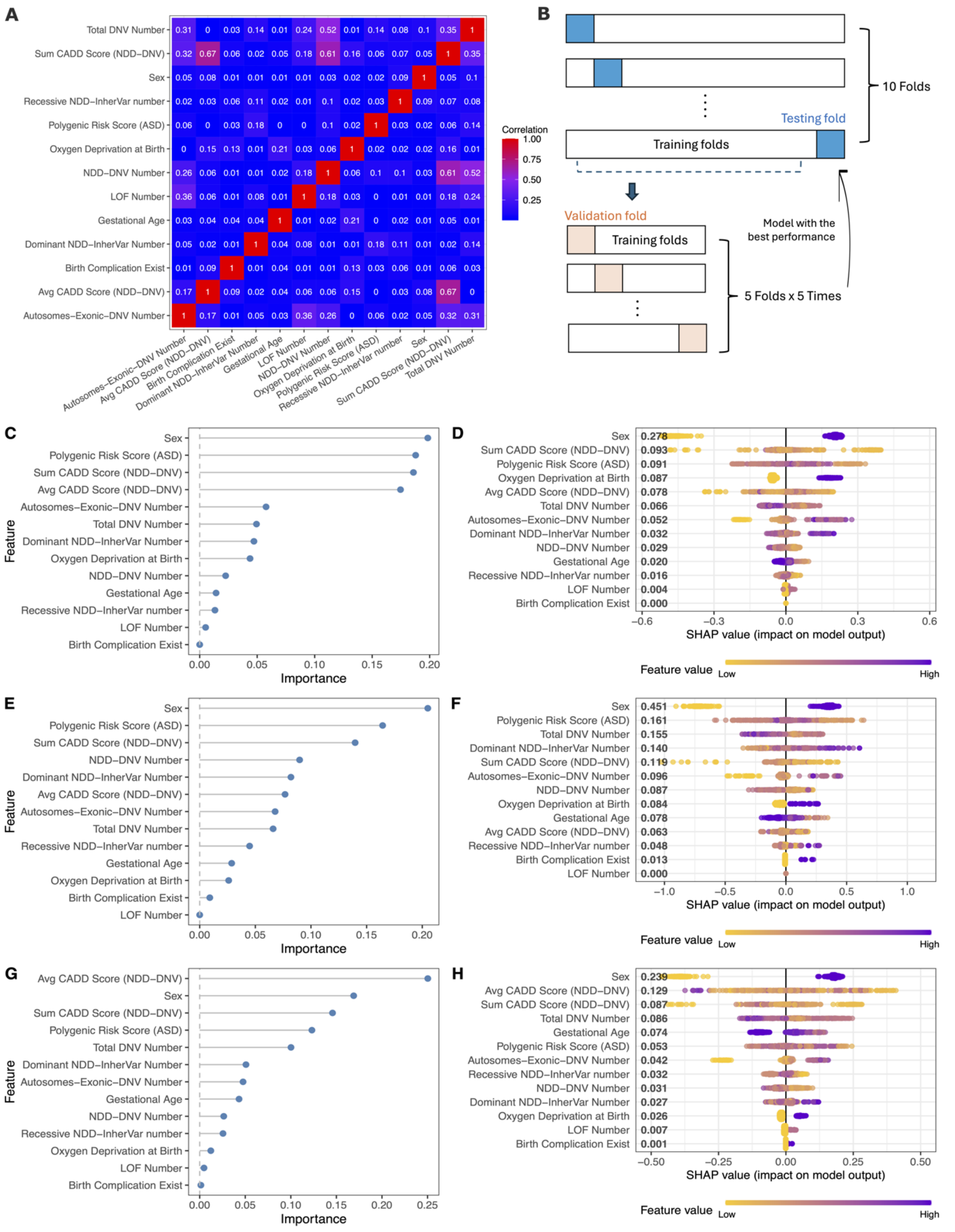

Fig S12. The design and evaluation of machine learning (ML) model. A. The correlation between features used in the ML model. B. The model training design. Feature importance (C, E, G) and SHAP values (D, F, H) for ML models trained with the XGBoost algorithm on the first three of the ten folds. C and D correspond to the first fold, E and F to the second fold, and G and H to the third fold.

Table S1: Description of diagnostic categories in SPARK cohort.

**Diagnostic category**	**Diagnostic sub-category**	**Variable**	**Definition**
behave	behave	behav_adhd	ADHD (Attention Deficit-Hyperactivity Disorder) or ADD
behave	behave	behav_conduct	Conduct Disorder
behave	behave	behav_intermitt_explos	Intermittent Explosive Disorder
behave	behave	behav_odd	Oppositional Defiant Disorder
growth	growth	growth_low_wt	Difficulty gaining weight
growth	growth	growth_macroceph	Large head size (macrocephaly)
growth	growth	growth_microceph	Small head size (microcephaly)
growth	growth	growth_obes	Obesity
growth	growth	growth_short	Short stature
mood	mood	mood_anx	Anxiety disorder, such as panic, phobia, agoraphobia, or generalized anxiety disorder (GAD) except for social anxiety
mood	mood	mood_bipol	Bipolar (Manic-Depressive) Disorder
mood	mood	mood_dep	Depression or dysthymia
mood	mood	mood_dmd	Disruptive Mood Dysregulation Disorder
mood	mood	mood_hoard	Hoarding
mood	mood	mood_ocd	Obsessive-Compulsive Disorder
mood	mood	mood_sep_anx	Separation Anxiety
mood	mood	mood_soc_anx	Social Anxiety Disorder/Social Phobia
sleep	sleep	sleep_dx	Sleep Disorder or sleep problem diagnosed by a professional
birth	birth_bone	birth_def_bone	Branching Question: Birth defects of bones, hands or feet
birth	birth_bone	birth_def_bone_club	Clubbed foot
birth	birth_bone	birth_def_bone_miss	Missing or malformed bones
birth	birth_bone	birth_def_bone_polydact	Extra fingers and/or extra toes
birth	birth_bone	birth_def_bone_spine	Spine deformity
birth	birth_cleft	birth_def_cleft_lip	Cleft lip
birth	birth_cleft	birth_def_cleft_palate	Cleft palate
birth	birth_cns	birth_def_cns	Branching Question: Brain and spinal cord birth defects
birth	birth_cns	birth_def_cns_brain	Brain malformation/abnormality (shown on MRI)
birth	birth_cns	birth_def_cns_myelo	Spina bifida/myelomeningocele (baby born with open spine, spinal cord outside of the body)
birth	birth_fac	birth_def_fac	Branching Question: Facial birth defect
birth	birth_gi	birth_def_gastro	Branching Question: Gastrointestinal (GI) birth defects

birth	birth_gi	birth_def_gi_esoph_atres	Esophageal atresia (no connection between the esophagus and stomach)
birth	birth_gi	birth_def_gi_hirschprung	Hirschsprung disease
birth	birth_gi	birth_def_gi_intest_malrot	Intestinal malrotation
birth	birth_gi	birth_def_gi_pylor_sten	Pyloric stenosis (blockage from stomach to small intestine)
birth	birth_thorac	birth_def_thorac	Branching Question: Heart or lung birth defect
birth	birth_thorac	birth_def_thorac_cdh	Congenital diaphragmatic hernia
birth	birth_thorac	birth_def_thorac_heart	Congenital heart disease/defect
birth	birth_thorac	birth_def_thorac_lung	Lung malformation
birth	birth_urogen	birth_def_urogen	Branching Question: Urinary or genital birth defect
birth	birth_urogen	birth_def_urogen_hypospad	Hypospadias (in boys, urinary opening is in the wrong place)
birth	birth_urogen	birth_def_urogen_renal	Kidney malformation (for example horseshoe kidney)
birth	birth_urogen	birth_def_urogen_renal_agen	Missing kidney
birth	birth_urogen	birth_def_urogen_uter_agen	Missing uterus
eat	eat	eating_disorder	Eating Disorder
eat	eat	feeding_dx	Feeding/eating problems
neuro	neuro	neuro_inf	Brain infection such as bacterial meningitis, encephalitis
neuro	neuro	neuro_lead	Lead poisoning
neuro	neuro	neuro_sz	Seizure disorder or epilepsy
neuro	neuro	neuro_tbi	Traumatic brain injury (hospitalized)
visaud	visaud	visaud_blind	Blindness
visaud	visaud	visaud_catar	Cataract
visaud	visaud	visaud_deaf	Deafness/hearing loss
visaud	visaud	visaud_strab	Strabismus
development	development	dev_lang_dis	Language delay or language disorder
development	development	dev_ld	Learning disability (LD, learning disorder, including reading, written expression, math, or NVLD (Nonverbal learning disability))
development	development	dev_motor	Motor delay (e.g., delay in walking) or developmental coordination disorder
development	development	dev_mutism	Mutism
development	development	dev_soc_prag	Social (Pragmatic) Communication Disorder
development	development	dev_speech	Speech articulation problems

Data comes from Basic Medical Screening dataset in SPARK phenotype dataset v9. The column Variable shows the variable name in original dataset. For birth complications, outcomes within one system were grouped as a single sub-diagnosis. For instance, if a child presented with both birth_def_cns_brain and birth_def_cns_myelo, we categorized this as a central nervous system (CNS) complication, treating it as one comorbidity within the birth category.

Table S2: Description and statistics of quantitative measures in SPARK cohort.

Measure	Full name	Variable	Description	ASD-preterm (n)	ASD-term (n)	Non-ASD-preterm (n)
CBCL_1-5	Child Behavior Checklist 1.5-5 years old	total_problems_t_score	Total problems T score	309	1681	0
CBCL_6-18	Child Behavior Checklist 6-18 years old	total_problems_t_score	Total problems T score	1101	7879	0
DCDQ	Developmental Coordination Disorder Questionnaire	final_score	Final score	4179	27829	0
RBS-R	Repetitive Behavior Scale-Revised	total_final_score	Total final score	5568	37653	0
SCQ	Social-Communication Questionnaire - Lifetime	final_score	Final score	7636	50527	2411
IQ	Intelligence Quotient	fsiq_score	Overall Full Scale IQ Score	415	2833	0

The column Variable shows the variable name in original dataset. The statistical numbers represent the individual counts with available data in each subgroup.

Table S3: Description of diagnostic categories in SSC cohort.

Diagnostic category	Dataset	Variable	Definition
birth	medhx_fam_birth_defects	abnormal_shape_polydactyly_proband	Abnormal shape of hands, feet, arms, or legs including Polydactyly - Proband
birth	medhx_fam_birth_defects	cleft_lip_palate_proband	Cleft lip/palate - Proband
birth	medhx_fam_birth_defects	congenital_heart_defect_proband	Congenital heart defect - Proband
birth	medhx_fam_birth_defects	kidney_defect_proband	Kidney defect - Proband
birth	medhx_fam_birth_defects	open_spine_proband	Open spine - Proband
birth	medhx_fam_birth_defects	other_birth_defect_proband	Other birth defect - Proband
development	medhx_fam_language	communication_disorder_proband	Communication Disorder, NOS - Proband
development	medhx_fam_language	expressive_lang_disorder_proband	Expressive Language Disorder - Proband
development	medhx_fam_language	mixed_expressive_disorder_proband	Mixed Expressive/Receptive Language Disorder - Proband
development	medhx_fam_language	other_lang_disorder_proband	Other Language Disorders - Proband
development	medhx_fam_language	pragmatics_lang_disorder_proband	Pragmatics Language Disorder - Proband
development	medhx_fam_language	receptive_lang_disorder_proband	Receptive Language Disorder - Proband
development	medhx_fam_language	reduced_articulation_proband	Reduced articulation/pronunciation - Proband
development	medhx_fam_language	speech_delay_proband	Speech delay requiring therapy - Proband
development	medhx_fam_language	stuttering_proband	Stuttering - Proband
development	medhx_fam_other_developmental	math_disorder_proband	Math Disorder - Proband
development	medhx_fam_other_developmental	mental_retardation_proband	Mental Retardation - Proband
development	medhx_fam_other_developmental	nonverbal_disorder_proband	Nonverbal Learning Disorder - Proband
development	medhx_fam_other_developmental	other_learning_delay_proband	Other Learning delay/disorder - Proband

development	medhx_fam_other_developmental	reading_disorder_proband	Reading Disorder - Proband
development	medhx_fam_other_developmental	written_expression_disorder_proband	Disorder of Written Expression - Proband
neuro	medhx_fam_neurological	cerebral_palsy_proband	Cerebral Palsy - Proband
neuro	medhx_fam_neurological	congenital_rubella_proband	Congenital Rubella - Proband
neuro	medhx_fam_neurological	cranial_nerve_disorder_proband	Cranial Nerve Disorder - Proband
neuro	medhx_fam_neurological	hydrocephalus_proband	Hydrocephalus - Proband
neuro	medhx_fam_neurological	landau_kleffner_syndrome_proband	Landau Kleffner Syndrome - Proband
neuro	medhx_fam_neurological	migraines_proband	Migraines - Proband
neuro	medhx_fam_neurological	neurofibromatosis_proband	Neurofibromatosis - Proband
neuro	medhx_fam_neurological	seizures_proband	Seizures - Proband
neuro	medhx_fam_neurological	tuberous_sclerosis_proband	Tuberous Sclerosis - Proband
behave	medhx_fam_psychiatric1	attention_deficit_proband	Attention Deficit Disorder/Attention Deficit Hyperactivity Disorder - Proband
behave	medhx_fam_psychiatric1	behavior_disorder_proband	Behavior Disorder - Proband
behave	medhx_fam_psychiatric1	other_psychotic_disorder_proband	Other Psychotic Disorders - Proband
behave	medhx_fam_psychiatric1	pica_proband	Pica - Proband
behave	medhx_fam_psychiatric1	tourettes_proband	Tourette's Disorder/Chronic Motor or vocal tic disorder/Tic disorder, NOS - Proband
mood	medhx_fam_psychiatric1	schizophrenia_proband	Schizophrenia - Proband
mood	medhx_fam_psychiatric2	adjustment_disorder_proband	Adjustment Disorder - Proband
mood	medhx_fam_psychiatric2	anxiety_disorder_proband	Anxiety Disorder - Proband
mood	medhx_fam_psychiatric2	bipolar_disorder_proband	Bipolar Disorder/Manic Depressive - Proband
mood	medhx_fam_psychiatric2	depressive_disorder_proband	Depressive Disorder - Proband
mood	medhx_fam_psychiatric2	dysthymic_disorder_proband	Dysthymic Disorder, Mood Disorder NOS - Proband

mood	medhx_fam_psychiatric2	obsessive_compulsive_proband	Obsessive Compulsive Disorder - Proband
mood	medhx_fam_psychiatric2	other_psych_disorder2_proband	Other Psych Disorder 2 - Proband
mood	medhx_fam_psychiatric2	personality_disorder_proband	Personality Disorder - Proband
mood	medhx_fam_psychiatric2	post_traumatic_stress_proband	Post Traumatic Stress Disorder - Proband
mood	medhx_fam_psychiatric2	social_phobia_proband	Social Phobia - Proband
eat	medhx_fam_psychiatric1	eating_disorder_proband	Eating Disorder - Proband
eat	medhx_perinatal	feeding_difficulty	Feeding difficulty
growth	medhx_perinatal	physical_anomalies_heart	Heart Defect
growth	medhx_perinatal	physical_anomalies_kidney	Kidney Defect
growth	medhx_perinatal	physical_anomalies_other	Other Defect
growth	medhx_perinatal	physical_anomalies_polydactyly	If yes specify: - Abnormal shape hands, feet, arms, or legs including Polydactyly
visaud	medhx_hearing_vision_allergies	hearing	Has a Hearing Problem
visaud	medhx_hearing_vision_allergies	vision	Has a Vision Problem?

Data comes from datasets listed in column Dataset in SSC phenotype dataset. The column Variable shows the variable name in original dataset.

Table S4: Description and statistics of quantitative measures in SSC cohort.

Measure	Full name	Variable	Description	ASD-preterm (n)	ASD-term (n)
CBCL_2-5	Child Behavior Checklist 1.5-5 years old	total_problems_t_score	Total Problems T Score	44	394
CBCL_6-18	Child Behavior Checklist 6-18 years old	total_problems_t_score	Total Problems T Score	111	1082
DCDQ	Developmental Coordination Disorder Questionnaire	total	Total score	153	1425
SCQ	Social-Communication Questionnaire - Lifetime	summary_score	Summary_score	136	1268
IQ	Intelligence Quotient	fs_deviation_score	Full Scale Deviation IQ Score	107	1156

The column Variable shows the variable name in original dataset. The statistical numbers represent the individual counts with available data in each subgroup.

Table S5: Quality control and parameters used in polygenic risk score calculation.

**Parameter used in Plink for QC**		
**Parameter**	**Threshold**	**Description**
--mind	0,04	Genotyping threshold of individuals (less than 3 standard deviations of mean missing call rate)
--geno	0,01	Missingness of variants
--hwe	1,00E-06	Hardy–Weinberg equilibrium of variants
--maf	0,01	Minor allele frequency threshold of variants
--indep-pairwise	200 50 0.25	Linkage disequilibrium of variants
--het		Individuals with heterozygosity rates out of the range of ±3 standard deviations of mean heterozygosity rate are removed
**Default Parameter used in PRScs**		
**Parameter**	**Threshold**	**Description**
--a	1	PARAM_A
--b	0,5	PARAM_B
--phi	0,01	PARAM_PHI
--n_iter	1000	MCMC_ITERATIONS
--n_burnin	500	MCMC_BURNIN
--thin	5	MCMC_THINNING_FACTOR

Table S6: Feature selected in machine learning model construction.

Feature	Description (data type)
Sex	Sex assigned at birth (female|male)
Birth complication Exist	Labeled as 1 if individual has any one of the birth complications (1|0)
Gestational at age	Gestational age at birth (number)
Oxygen Deprivation at Birth	Labeled as 1 if individual had insufficient oxygen at birth with NICU stay (1|0)
Polygenic Risk Score (ASD)	Standardized ASD Polygenic Risk Score (number)
Recessive NDD-InherVar Number	The number of inherited variants on exonic recessive neurodevelopmental disorder (NDD) -related genes (number)
Dominant NDD-InherVar Number	The number of inherited variants on exonic dominant NDD genes (number)
Total DNV Number	The number of de novo variants (DNV) (number)
NDD-DNV Number	The number of DNV on NDD genes (number)
Autosomes-Exonic-DNV Number	The number of DNV on autosomes and only considering protein-coding genes (number)
Sum CADD Score (NDD-DNV)	The sum of the Combined Annotation Dependent Depletion (CADD) scores of DNV on NDD genes (number)
Avg CADD Score (NDD-DNV)	The average of CADD scores of DNV on NDD genes (number)
LOF Number	The number of LOF DNV (number)

* Variants mentioned in this table were identified in genome sequencing.

Table S7: The values of hyperparameter tuning for machine learning models.

Algorithm	Parameter	Value
XGBoost	nrounds	100, 200, 300, 400
	eta	0.03, 0.05, 0.1, 0.2, 0.3
	max_depth	3, 6, 9, 12
	gamma	0, 1, 3, 5
	colsample_bytree	0.3, 0.5, 0.75
	min_child_weight	1, 3
	subsample	0,8
	scale_pos_weight	0,6
Random Forest	mtry	1, 3, 5, 7, 10, 15, 20
	ntree	1000
	nodesize	1
Linear SVM	C	0, 0.01, 0.05, 0.1, 0.25, 0.5, 0.75, 1, 1.25, 1.5, 1.75, 2
	preProcess	"center", "scale"

Table S8: The prevalence and odds ratio of diagnostic categories compared between ASD-preterm and ASD-term in the SPARK cohort.

Diagnostic category	Phenotype	Diagnosed number	Prevalence	OR (95% CI)	FDR-p	GEE: OR (95% CI)	GEE: FDR-p
Behavior	Preterm	3900	0.42	1.19 (1.14–1.25)	5.04E-15	1.27 (1.21–1.33)	3.16E-24
	Term	24809	0.38				
Birth	Preterm	529	0.06	2.18 (1.97–2.4)	5.61E-55	2.25 (2.03–2.49)	1.20E-54
	Term	1774	0.03				
Development	Preterm	6597	0.72	1.5 (1.43–1.57)	4.88E-61	2.45 (2.27–2.63)	2.37E-43
	Term	40896	0.63				
Eat	Preterm	2070	0.23	1.71 (1.62–1.8)	4.12E-86	1.67 (1.58–1.77)	2.43E-75
	Term	9456	0.15				
Growth	Preterm	1748	0.19	2.18 (2.06–2.31)	9.24E-158	2.23 (2.1–2.37)	1.37E-150
	Term	6310	0.10				
Mood	Preterm	3245	0.35	1.1 (1.05–1.15)	4.39E-05	1.31 (1.25–1.38)	7.80E-27
	Term	21548	0.33				
Neuro	Preterm	923	0.10	1.64 (1.52–1.76)	1.61E-38	1.75 (1.62–1.89)	4.41E-46
	Term	4150	0.06				
Sleep	Preterm	2417	0.26	1.45 (1.38–1.52)	3.44E-47	1.49 (1.42–1.57)	2.25E-51
	Term	12856	0.20				
Visaud	Preterm	944	0.10	2.02 (1.87–2.17)	3.88E-76	2.15 (1.99–2.33)	5.50E-84
	Term	3493	0.05				

FDR-p values are adjusted p-values by False Discovery Rate method. ORs with 95% CI are given among ASD individuals born preterm vs term. Both unadjusted odds ratios and those adjusted for covariates (sex and age) and family clustering using GEE models are provided. Diagnostic categories are described in table S1.

Table S9: Statistical test of differences of prevalence between different preterm stages (For SPARK).

Diagnosis	Comparison	p.Chisq	p.adj.Chisq (FDR adjustment)	p.adj.tau (FDR adjustment)
Behave	Extremely preterm : Very preterm	0,404	0,577	0,3
Behave	Extremely preterm : Moderate preterm	0,817	0,817	
Behave	Extremely preterm : Late preterm	0,651	0,723	
Behave	Extremely preterm : Term	0,0154	0,0385	
Behave	Very preterm : Moderate preterm	0,473	0,591	
Behave	Very preterm : Late preterm	0,0842	0,168	
Behave	Very preterm : Term	2,83E-05	0,000142	
Behave	Moderate preterm : Late preterm	0,349	0,577	
Behave	Moderate preterm : Term	0,000264	0,00088	
Behave	Late preterm : Term	1,07E-07	1,07E-06	
Growth	Extremely preterm : Very preterm	0,0518	0,0576	0,038
Growth	Extremely preterm : Moderate preterm	0,0104	0,0149	
Growth	Extremely preterm : Late preterm	3,33E-07	6,66E-07	
Growth	Extremely preterm : Term	9,01E-48	4,51E-47	
Growth	Very preterm : Moderate preterm	0,577	0,577	
Growth	Very preterm : Late preterm	0,00343	0,00572	
Growth	Very preterm : Term	2,91E-38	7,28E-38	
Growth	Moderate preterm : Late preterm	0,0141	0,0176	
Growth	Moderate preterm : Term	1,79E-41	5,97E-41	
Growth	Late preterm : Term	9,31E-89	9,31E-88	
Mood	Extremely preterm : Very preterm	0,0298	0,093	0,92
Mood	Extremely preterm : Moderate preterm	0,497	0,621	
Mood	Extremely preterm : Late preterm	0,366	0,523	
Mood	Extremely preterm : Term	0,914	0,914	

Mood	Very preterm : Moderate preterm	0,0737	0,147	
Mood	Very preterm : Late preterm	0,0372	0,093	
Mood	Very preterm : Term	0,000387	0,00387	
Mood	Moderate preterm : Late preterm	0,888	0,914	
Mood	Moderate preterm : Term	0,199	0,332	
Mood	Late preterm : Term	0,0033	0,0165	
Sleep	Extremely preterm : Very preterm	0,372	0,413	1
Sleep	Extremely preterm : Moderate preterm	0,0544	0,109	
Sleep	Extremely preterm : Late preterm	0,271	0,339	
Sleep	Extremely preterm : Term	0,0027	0,00675	
Sleep	Very preterm : Moderate preterm	0,268	0,339	
Sleep	Very preterm : Late preterm	0,991	0,991	
Sleep	Very preterm : Term	4,30E-07	1,43E-06	
Sleep	Moderate preterm : Late preterm	0,13	0,217	
Sleep	Moderate preterm : Term	3,54E-14	1,77E-13	
Sleep	Late preterm : Term	2,64E-31	2,64E-30	
Birth	Extremely preterm : Very preterm	0,425	0,531	0,3
Birth	Extremely preterm : Moderate preterm	0,733	0,733	
Birth	Extremely preterm : Late preterm	0,208	0,297	
Birth	Extremely preterm : Term	6,66E-10	1,66E-09	
Birth	Very preterm : Moderate preterm	0,577	0,641	
Birth	Very preterm : Late preterm	0,00678	0,0136	
Birth	Very preterm : Term	3,46E-20	1,73E-19	
Birth	Moderate preterm : Late preterm	0,0301	0,0502	
Birth	Moderate preterm : Term	8,51E-20	2,84E-19	
Birth	Late preterm : Term	1,18E-30	1,18E-29	
Eat	Extremely preterm : Very preterm	0,00608	0,00869	0,038
Eat	Extremely preterm : Moderate preterm	0,000225	0,000375	

Eat	Extremely preterm : Late preterm	2,69E-08	5,38E-08	
Eat	Extremely preterm : Term	2,27E-33	1,14E-32	
Eat	Very preterm : Moderate preterm	0,386	0,386	
Eat	Very preterm : Late preterm	0,022	0,0275	
Eat	Very preterm : Term	3,14E-19	1,05E-18	
Eat	Moderate preterm : Late preterm	0,183	0,203	
Eat	Moderate preterm : Term	8,31E-18	2,08E-17	
Eat	Late preterm : Term	4,45E-44	4,45E-43	
Neuro	Extremely preterm : Very preterm	0,0213	0,0237	0,038
Neuro	Extremely preterm : Moderate preterm	0,00549	0,00686	
Neuro	Extremely preterm : Late preterm	2,44E-09	4,88E-09	
Neuro	Extremely preterm : Term	3,05E-26	3,05E-25	
Neuro	Very preterm : Moderate preterm	0,713	0,713	
Neuro	Very preterm : Late preterm	0,00162	0,0027	
Neuro	Very preterm : Term	3,55E-14	8,88E-14	
Neuro	Moderate preterm : Late preterm	0,0032	0,00457	
Neuro	Moderate preterm : Term	4,16E-15	1,39E-14	
Neuro	Late preterm : Term	8,27E-16	4,14E-15	
Visaud	Extremely preterm : Very preterm	7,46E-07	1,07E-06	0,038
Visaud	Extremely preterm : Moderate preterm	3,30E-09	5,50E-09	
Visaud	Extremely preterm : Late preterm	1,64E-25	5,47E-25	
Visaud	Extremely preterm : Term	1,26E-77	1,26E-76	
Visaud	Very preterm : Moderate preterm	0,493	0,493	
Visaud	Very preterm : Late preterm	0,000268	0,000335	
Visaud	Very preterm : Term	6,68E-23	1,67E-22	
Visaud	Moderate preterm : Late preterm	0,00256	0,00284	
Visaud	Moderate preterm : Term	3,35E-22	6,70E-22	
Visaud	Late preterm : Term	4,49E-28	2,24E-27	

Development	Extremely preterm : Very preterm	0,00168	0,0028	0,15
Development	Extremely preterm : Moderate preterm	0,00915	0,0131	
Development	Extremely preterm : Late preterm	1,66E-05	3,32E-05	
Development	Extremely preterm : Term	7,82E-18	3,91E-17	
Development	Very preterm : Moderate preterm	0,441	0,49	
Development	Very preterm : Late preterm	0,514	0,514	
Development	Very preterm : Term	5,27E-09	1,32E-08	
Development	Moderate preterm : Late preterm	0,0772	0,0965	
Development	Moderate preterm : Term	1,50E-14	5,00E-14	
Development	Late preterm : Term	2,87E-33	2,87E-32	

Kendall's rank tau test was performed with ranking from extremely preterm to term order, and p-values were adjusted by FDR method.

Table S10: The prevalence and odds ratio of multimorbidity in diagnostic categories compared between ASD-preterm and ASD-term in the SPARK cohort.

Multimorbidity	Phenotype	Diagnosed number	Prevalence	OR (95% CI)	FDR-p	GEE: OR (95% CI)	GEE: FDR-p
0	Preterm	766	0.08	0.60 (0.55–0.65)	3.32E-39	0.57 (0.53–0.62)	4.02E-43
	Term	8585	0.13				
1	Preterm	2438	0.27	0.73 (0.70–0.77)	8.22E-35	0.70 (0.67–0.74)	3.14E-43
	Term	21421	0.33				
2	Preterm	2076	0.23	0.93 (0.88–0.98)	0.004	0.95 (0.90–0.997)	0.039
	Term	15559	0.24				
3	Preterm	1695	0.18	1.19 (1.13–1.26)	1.10E-09	1.24 (1.17–1.31)	2.48E-13
	Term	10349	0.16				
4	Preterm	1084	0.12	1.48 (1.38–1.59)	3.61E-29	1.55 (1.44–1.66)	1.08E-33
	Term	5369	0.08				
>=5	Preterm	1137	0.12	2.31 (2.16–2.48)	3.59E-126	2.45 (2.27–2.63)	8.96E-127
	Term	3738	0.06				

FDR-p values are adjusted p-values by False Discovery Rate method. ORs with 95% CI are given among ASD individuals born preterm vs term when focusing on each multimorbidity number. Both unadjusted odds ratios and those adjusted for covariates (sex and age) and family clustering using GEE models are provided. Diagnostic categories are described in table S1.

Table S11: FDR-adjusted p values in Post-hoc comparison of Chi-square test for the burden of multimorbidities in different groups (for SPARK).

**Number of multimorbidities**	**0**	**1**	**2**	**3**	**4**	**>=5**
Whole preterm vs Term	6,63E-39	2,47E-34	0,026	5,48E-09	1,44E-28	3,59E-126
Extremely preterm vs Very preterm	0,46	1	1	1	1	0,33
Extremely preterm vs Moderate preterm	0,014	1	1	0,30	1	0,36
Extremely preterm vs Late preterm	0,002	0,01	1	0,098	1	7,99E-06
Extremely preterm vs Term	2,33E-10	5,92E-09	0,64	0,0003	0,0002	3,27E-40
Very preterm vs Moderate preterm	0,92	1	1	0,64	1	1
Very preterm vs Late preterm	0,33	0,13	1	0,22	1	0,060
Very preterm vs Term	1,85E-08	2,70E-08	0,37	0,0003	2,16E-06	6,41E-29
Moderate preterm vs Late preterm	1	0,21	1	1	1	0,012
Moderate preterm vs Term	2,91E-06	3,63E-09	0,63	0,28	6,96E-07	1,14E-38
Late preterm vs Term	3,08E-22	1,78E-15	0,25	0,0007	2,52E-18	1,14E-63

The multimorbidity counts the number of diagnostic categories described in Table S1.

Table S12: The prevalence and odds ratio of diagnostic categories compared between ASD-preterm and non-ASD-preterm in the SPARK cohort.

Diagnostic category	Phenotype	Diagnosed number	Prevalence	OR (95% CI)	FDR-p	GEE: OR (95% CI)	GEE: FDR-p
Behavior	ASD	3900	0.42	3.03 (2.73–3.36)	1.04E-103	2.43 (2.19–2.71)	3.12E-103
	non-ASD	529	0.20				
Birth	ASD	529	0.06	1.93 (1.53–2.45)	2.71E-08	1.78 (1.4–2.27)	2.71E-08
	non-ASD	83	0.03				
Development	ASD	6597	0.72	8.77 (7.94–9.71)	0	9.1 (8.19–10.1)	0
	non-ASD	607	0.22				
Eat	ASD	2070	0.23	5.57 (4.67–6.7)	6.73E-95	5.88 (4.91–7.05)	1.51E-94
	non-ASD	134	0.05				
Growth	ASD	1748	0.19	2.02 (1.77–2.32)	1.00E-25	2.02 (1.76–2.32)	1.29E-25
	non-ASD	281	0.10				
Mood	ASD	3245	0.35	3 (2.68–3.36)	1.18E-86	2.65 (2.36–2.99)	2.12E-86
	non-ASD	416	0.15				
Neuro	ASD	923	0.10	4.13 (3.26–5.33)	1.64E-34	3.9 (3.03–5.02)	2.45E-34
	non-ASD	71	0.03				
Sleep	ASD	2417	0.26	6.94 (5.82–8.36)	8.40E-126	6.87 (5.72–8.24)	3.78E-125
	non-ASD	132	0.05				
Visaud	ASD	944	0.10	2.23 (1.85–2.7)	8.68E-18	2.25 (1.85–2.73)	9.77E-18
	non-ASD	132	0.05				

FDR-p values are adjusted p-values by False Discovery Rate method. ORs with 95% CI are given among ASD vs non-ASD individuals born preterm. Both unadjusted odds ratios and those adjusted for covariates (sex and age) and family clustering using GEE models are provided. Diagnostic categories are described in table S1. Totally 9196 ASD and 2706 non-ASD.

Table S13: The prevalence and odds ratio of multimorbidity in diagnostic categories compared between ASD-preterm and non-ASD-preterm in the SPARK cohort.

Multimorbidity	Phenotype	Diagnosed number	Prevalence	OR (95% CI)	FDR-p	GEE: OR (95% CI)	GEE: FDR-p
0	ASD	766	0.08	0.09 (0.08–0.1)	0	0.1 (0.09–0.11)	0
	Non-ASD	1357	0.50				
1	ASD	2438	0.27	0.92 (0.84–1.02)	0.097	0.98 (0.88–1.08)	0.097
	Non-ASD	761	0.28				
2	ASD	2076	0.23	2.04 (1.81–2.31)	2.31E-30	1.92 (1.69–2.18)	2.31E-30
	Non-ASD	338	0.12				
3	ASD	1695	0.18	4.51 (3.76–5.45)	6.45E-67	4.16 (3.45–5.01)	6.45E-67
	Non-ASD	129	0.05				
4	ASD	1084	0.12	5.17 (4.07–6.7)	1.76E-46	4.89 (3.8–6.3)	1.76E-46
	Non-ASD	68	0.03				
>=5	ASD	1137	0.12	7.04 (5.38–9.42)	2.37E-56	6.59 (4.97–8.74)	2.37E-56
	Non-ASD	53	0.02				

FDR-p values are adjusted p-values by False Discovery Rate method. ORs with 95% CI are given among ASD vs non-ASD individuals born preterm when focusing on each multimorbidity number. Both unadjusted odds ratios and those adjusted for covariates (sex and age) and family clustering using GEE models are provided. Diagnostic categories are described in table S1. Totally 9196 ASD and 2706 non-ASD. Totally 157 preterm and 1479 term.

Table S14: The prevalence and odds ratio of diagnostic categories compared between ASD-preterm and ASD-term in the SSC cohort

Diagnostic category	Phenotype	Diagnosed number	Prevalence	OR (95% CI)	FDR-p	GEE: OR (95% CI)	GEE: FDR-p
Behavior	Preterm	32	0.20	1.45 (0.95–2.18)	0.21	1.54 (1.01–2.35)	0.12
	Term	222	0.15				
Birth	Preterm	5	0.03	0.8 (0.27–1.84)	0.68	0.78 (0.31–1.98)	0.75
	Term	60	0.04				
Development	Preterm	79	0.50	0.94 (0.68–1.31)	0.73	0.94 (0.67–1.3)	0.75
	Term	766	0.52				
Eat	Preterm	53	0.34	2.58 (1.79–3.68)	7.7E-07	2.55 (1.78–3.66)	2.85E-06
	Term	244	0.16				
Growth	Preterm	14	0.09	2.02 (1.06–3.58)	0.08	1.98 (1.08–3.62)	0.10
	Term	69	0.05				
Mood	Preterm	7	0.04	0.8 (0.33–1.65)	0.68	0.88 (0.39–1.96)	0.75
	Term	83	0.06				
Neuro	Preterm	8	0.05	0.75 (0.33–1.47)	0.68	0.76 (0.36–1.59)	0.75
	Term	101	0.07				
Visaud	Preterm	35	0.22	1.15 (0.77–1.7)	0.68	1.27 (0.84–1.92)	0.53
	Term	295	0.20				

FDR-p values are adjusted p-values by False Discovery Rate method. ORs with 95% CI are given among ASD individuals born preterm vs term. Both unadjusted odds ratios and those adjusted for covariates (sex and age) and family clustering using GEE models are provided. Diagnostic categories are described in table S1. Totally 157 preterm and 1479 term.

Table S15: The prevalence and odds ratio of multimorbidity in diagnostic categories compared between ASD-preterm and ASD-term in the SSC cohort.

Multimorbidity	Phenotype	Diagnosed number	Prevalence	OR (95% CI)	FDR-p	GEE: OR (95% CI)	GEE: FDR-p
0	Preterm	31	0.20	0.8 (0.52–1.19)	0.34	0.78 (0.51–1.18)	0.30
	Term	350	0.24				
1	Preterm	57	0.36	0.77 (0.54–1.08)	0.21	0.75 (0.53–1.05)	0.16
	Term	631	0.43				
2	Preterm	41	0.26	1.18 (0.8–1.7)	0.40	1.22 (0.83–1.78)	0.31
	Term	342	0.23				
3	Preterm	19	0.12	1.71 (0.99–2.8)	0.18	1.76 (1.04–2.97)	0.14
	Term	111	0.08				
>=4	Preterm	9	0.06	1.96 (0.88–3.92)	0.18	2.05 (0.98–4.29)	0.14
	Term	45	0.03				

FDR-p values are adjusted p-values by False Discovery Rate method. ORs with 95% CI are given among ASD individuals born preterm vs term when focusing on each multimorbidity number. Both unadjusted odds ratios and those adjusted for covariates (sex and age) and family clustering using GEE models are provided. Diagnostic categories are described in table S1. Totally 157 preterm and 1479 term.

Table S16: P-values from GEE models for the correlation between multimorbidity and DNV count (SPARK cohort).

	GS_DNV	GS_LOF	GS_DNV_NDD	GS_LOF_NDD	ES_DNV	ES_LOF	ES_DNV_NDD	ES_LOF_NDD
**Model 1: multimorbidity ~ Variant_count + sex, data = Whole**								
Variant_count	0.16 (-)	0.037 (+)	0,2	5.3e-06 (+)	1.1e-05 (+)	1.9e-09 (+)	6.6e-14 (+)	<2e-16 (+)
**Model 2: Preterm ~ Variant_count * multimorbidity + sex, data = ASD**								
Variant_count	0,6	0,904	0,217	0,71	0,68	0,98	0,36	0,34
multimorbidity	0,91	5.5e-07 (+)	0,54	4.9e-07 (+)	3.9e-07 (+)	9.2e-14 (+)	5.6e-12 (+)	1e-14 (+)
Variant_count:multimorbidity	0,22	0,904	0.040 (+)	0,61	0,98	0,97	0,84	0,66
**Model 3: ASD ~ Variant_count * multimorbidity + sex, data = Preterm**								
Variant_count	0,053	0,59	0.0066 (-)	0,596	0.049 (+)	0,59	0,58	0,48
multimorbidity	0,77	9.8e-12 (+)	0,33	1.5e-12 (+)	8.6e-08 (+)	2.2e-12 (+)	1.1e-10 (+)	1.1e-13 (+)
Variant_count:multimorbidity	0,22	0,18	0.0325 (+)	0,424	0,47	0,26	0,85	0,92

The +/- after p value reflects the positive/negative estimation of variables. GS = genome sequencing, ES = exome sequencing, NDD = NDD-related genes.
